# Understanding a high-risk acute myeloid leukemia by analyzing the interactome of its major driver mutation

**DOI:** 10.1371/journal.pgen.1010463

**Published:** 2022-10-26

**Authors:** Claudia Chiriches, Nathalie Nicolaisen, Maria Wieske, Heba Elhaddad, Ecmel Mehmetbeyoglu, Caroline Alvares, Dörte Becher, Paul Hole, Oliver Gerhard Ottmann, Martin Ruthardt

**Affiliations:** 1 Division of Cancer and Genetics, Section of Hematology, School of Medicine, Cardiff University, Cardiff, United Kingdom; 2 Experimental Clinical Medical Center (ECMC) Cardiff, School of Medicine, Cardiff University, Cardiff, United Kingdom; 3 Department of Hematology, Medical Clinic II Goethe University Frankfurt, Germany; 4 Faculty of Medicine, Department of Clinical Pathology, Mansoura University, Mansoura, Egypt; 5 Institute of Microbiology, Microbial Proteomics, Ernst Moritz Arndt University, Greifswald, Germany; Brigham and Women’s Hospital, UNITED STATES

## Abstract

The WHO classifies t(6;9)-positive acute myeloid leukemia (AML) as a subgroup of high-risk AML because of its clinical and biological peculiarities, such as young age and therapy resistance. t(6;9) encodes the DEK/NUP214 fusion oncoprotein that targets only a small subpopulation of bone marrow progenitors for leukemic transformation. This distinguishes DEK/NUP214 from other fusion oncoproteins, such as PML/RARα, RUNX1/ETO, or MLL/AF9, which have a broad target population they block differentiation and increase stem cell capacity. A common theme among most leukemogenic fusion proteins is their aberrant localization compared to their wild-type counterparts. Although the actual consequences are widely unknown, it seems to contribute to leukemogenesis most likely by a sequester of interaction partners. Thus, we applied a global approach to studying the consequences of the aberrant localization of t(6;9)-DEK/NUP214 for its interactome. This study aimed to disclose the role of localization of DEK/NUP214 and the related sequester of proteins interacting with DEK/NUP214 for the determination of the biology of t(6;9)-AML. Here we show the complexity of the biological consequences of the expression of DEK/NUP214 by an in-depth bioinformatic analysis of the interactome of DEK/NUP214 and its biologically dead mutants. DEK/NUP214’s interactome points to an essential role for aberrant RNA-regulation and aberrant regulation of apoptosis and leukocyte activation as a significant determinant of the phenotype of t(6;9)-AML. Taken together, we provide evidence that the interactome contributes to the aberrant biology of an oncoprotein, providing opportunities for developing novel targeted therapy approaches.

## Author summary

The frequency of acute myeloid leukemia (AML), the most common acute leukemia in adults, increases with age while its outcome worsens. Risk stratification subdivides AML patients into good, intermediate, and poor risk groups concerning their overall survival (OS). The presence of tumor-specific genetic modifications and mutated proteins they encode influences the OS of a patient. Unfortunately, their role in OS and therapy response is not well understood. Here, we started with the commonalities most of these disease-defining modifications share. One is their aberrant localization within the tumor cells, accompanied by the changed localization of their interactor proteins. By studying all proteins interacting with one AML-defining mutated protein, we better understood the signaling pathways it disturbs. We found that the mutated protein studied, apart from programmed cell death and activation of white blood cells, strongly influences RNA-regulation through its interactors. RNA is transcribed from the genetic information encoded in the DNA and is responsible by many different mechanisms for correctly translating this information into proteins. Our findings define both new biomarkers and novel targets of molecular therapy. We expect to contribute to overcoming therapy resistance and thereby improve the overall survival of AML patients, particularly in the poor risk group.

## Introduction

Acute myeloid leukemia (AML)-inducing oncoproteins, such as PML/RARα (promyelocytic leukemia/retinoic acid receptor alpha), PLZF/RARα (promyelocytic leukemia zinc finger/retinoic acid receptor alpha), MLL/AF9 (mixed lineage leukemia/AF9), CBFβ/SMMHC (core binding factor beta/smooth-muscle myosin heavy-chain), and RUNX1/ETO (runt-related transcription factor 1/eight-twenty-one), encoded by the disease-defining translocations t(15;17), t(11;17), t(9;11), inv(16) and t(8;21), respectively, follow common themes in the processes underlying the induction of AML [1].

One of these common themes is the mislocalization of each translocation partner compared to its physiological counterpart. The consequence of such a mislocalization is the disruption of specific sub-nuclear compartments considered to be the cause of stage-specific differentiation block with enhanced cell survival or increased proliferation of bone marrow (BM) cells and aberrant self-renewal in early BM progenitors and leukemic stem cells [1].

Translocation does not only lead to mislocalization of the fusion partners but also of their interaction partners, as exemplified by t(15;17)-PML/RARα and its interaction with PU.1 (Purine-rich Box-1) and VDR (vitamin D3 receptor) [2,3]. t(15;17) disturbs the function of the translocation partners PML and RARα together with all related interaction partners within subcellular compartments. RARα exhibits a diffuse nuclear localization in normal cells, and PML localizes in the so-called PML-nuclear bodies in nuclear speckles. In leukemic cells, PML/RARα exhibits a microspeckled nuclear pattern. All-trans retinoic acid or arsenic trioxide (ATO), both used in the therapy of t(15;17)-positive AML, revert the PML/RARα localization pattern [4,5,6,7]. The response to ATO in t(15;17)-AML confirms the role of these leukemogenic mechanisms based on mislocalization and sequester [8]. ATO directly targets PML/RARα and triggers its degradation. Thus, the localization of the physiological counterparts, PML and RARα, can normalize [8]. In contrast, t(11;17)-AMLs are resistant to ATO because PLZF/RARα is not degraded [6,9].

As other fusion oncoproteins, such as RUNX1/ETO, behave similarly [10], translocation products may subsume the function of an otherwise compulsory combination of driver mutations found in normal karyotype AML [11]. Unfortunately, no therapeutic option of molecular targeting is available for any other AML-inducing oncoprotein than PML/RARα. Acknowledging the current impossibility of targeting AML-inducing oncoproteins, we took an alternative approach by trying to disclose those factors affected by the mislocalization of translocation partners in high-risk AML. We selected the t(6;9)-AML with its DEK/NUP214 fusion oncoprotein as a proof of principle.

The t(6;9) defines a distinct AML-subtype within the World Health Organization (WHO) classification because of its particular features and unmet clinical needs [12]. Patients harboring t(6;9) are younger than the median age of AML patients [13,14] and have a poor prognosis, with a median survival of only about one year [15,16]. Complete remission (CR) rates do not exceed 50%, and the only curative approach for t(6;9)-AML is hematopoietic stem cell transplantation during the first CR [17]. At diagnosis, t(6;9) is the only structural chromosomal aberration with DEK/nuclear pore complex 214 (DEK/NUP214) being the only gene product of t(6;9) [13] suggesting a decisive role for DEK/NUP214 in leukemia initiation and maintenance [18]. However, the mechanisms by which DEK/NUP214 transforms cells and mediates therapy resistance are still widely unknown.

Here we investigated the localization of DEK/NUP214 compared to wild-type (wt) DEK and wt NUP214 (also known as CAN), respectively. First, we wanted to understand the impact of the mislocalization of DEK/NUP214 on its interactions with other proteins. Therefore, we studied its interactome using Tandem affinity purification (TAP)-tag proteomics, and we disclosed those complexes which DEK/NUP214 influences. In a second step, we wanted to evidence those interactors relevant for the induction of the DEK/NUP214 phenotype and separate them from those that were supposedly irrelevant. Thus, we compared the interactome of DEK/NUP214 with that of its functionally inactive mutants.

These studies disclosed biological processes directly influenced by DEK/NUP214 that may play a role in the DEK/NUP214 induced leukemic phenotype.

## Materials and methods

### Plasmids, Oligos

See S1 Data

### Cell lines and cell culture

FKH1, U937, and 293T cells were obtained from the German Collection of Microorganisms and Cell Cultures (DSMZ), Braunschweig, Germany. U937-DEK/NUP214 were already described elsewhere [18]. We kept FKH1 and U937 cells in RPMI-1640 medium supplemented with 20% and 10% fetal calf serum (FCS)(Invitrogen). In addition, we cultured 293T in Dulbecco’s modified Eagle medium (DMEM) supplemented with 10% FCS. Transfection of 293T cells was performed by calcium-phosphate precipitation according to widely established protocols. 48h after transfection, the cells were lysed for further use.

### Animals

All animal studies were performed following international animal protection guidelines and approved by the regulating institution for the animal facility at the Goethe University Frankfurt (Regierungspräsidium Darmstadt—approval number F39/08). All mice were kept under pathogen-free conditions with controlled temperature and humidity conditions (21 ± 1°C, 40–60%), exposed to regular 12h light/12h darkness cycles, and with access to food and water ad libitum.

### Isolation of stem cell antigen (Sca1)+/lin- hematopoietic stem and progenitor cells (HSPCs)

Sca1+/lin- HSPCs were isolated from 8 to 12-week-old female C57BL/6N mice (Janvier, St. Berthevin, France). The cells were “lineage depleted” by labeling the cells with biotin-conjugated lineage panel antibodies against B220, CD3e, Gr-1, Mac-1, and Ter-119 (Miltenyi, Bergisch-Gladbach, Germany). According to manufacturer’s instructions (Miltenyi), we removed labeled cells using “MACS” cell separation columns. Finally, we purified Sca1+/lin- HSPCs cells by Sca1+ immunomagnetic beads using again “MACS” cell separation columns. Prior to further use, the purified cells were pre-stimulated in medium containing mIL-3 (20 ng/mL), mIL-6 (20 ng/mL) and mSCF (100 ng/mL)(Cell Concepts).

### Primary cell culture, colony-forming unit-day 12 (CFU-S12)

We performed CFU-S12 assays as described previously [19]. Briefly, Sca1+/Lin- cells were isolated from the C57BL/6N mice and pre-stimulated for two days before retroviral transduction and cultivation for ten days in DMEM medium supplemented with 10% FCS, mIL-6, mSCF, and mIL-3. Then, 48h after transduction, GFP was measured by flow cytometry (BD Fortessa) as a readout for transduction efficiency. The cells were harvested and inoculated into lethally irradiated (11 Gy) female recipient mice. The inoculated mice were culled 12 days later, and the spleens were fixed in Tellysniczky’s fixative for colony counting and photographed.

### Proteomics

#### Stable isotope labeling by amino acids in cell culture (SILAC) labeling

For complete labeling, cells were grown in SILAC medium (DMEM supplemented with 10% FCS dialyzed to eliminate the unlabeled a.a. (Gibco), 1% L-Glutamine, 1% Pen/Strep, essential amino acids) for at least five days or five divisions [21]. We used arginine and lysine (1%) enriched in stable isotopes of 13C and 15N (SILAC heavy—Svy) to label the cells before transfection with the empty vector control (TAP-). Unmodified a.a. (SILAC light -Sli) were used for the cells to be transfected with a vector encoding TAP-DEK/NUP214 or TAP-tagged mutants.

#### Tandem affinity purification (TAP)-tag

48 hours after transfection, we lysed 293T cells and precipitated TAP-tagged proteins in two consecutive affinity purification steps. In a first step, we precipitated the TAP-tagged proteins exploiting the interaction between IgG bound to Sepharose beads and the two Protein A sequences of the TAP-tag. The resulting complexes were subjected to Tobacco Etch Virus (TEV)protease cleavage at ENLYFQ to eliminate the first set of unspecific interactors. The second precipitation used the interaction between the streptavidin binding protein of the TAP-tag and streptavidin beads eluting the final complex by biotin competition. The eluted proteins from the Svy control cells were mixed 1:1 with each eluted light-labeled sample protein. These samples were run on an SDS-PAGE, subjected to in-gel trypsin digestion, and the resulting peptides were sequenced. MS and MS/MS data were acquired with an LTQ-Orbitrap mass spectrometer (Thermos Fisher Scientific) online coupled to the LC system.

#### Mass spectrometry (MS) data processing

All MS files were processed with MaxQuant version 1.6.14.0, and the peptides were searched against the UniProt (uniprot.org) human Database using the Andromeda search engine integrated into MaxQuant. Default parameters were applied except described otherwise. Methionine oxidation was set as variable modification and carbamidomethylation of cysteine as fixed modification. We used trypsin as a digestion enzyme and set the minimum peptide length at 7 amino acids with a maximum of 2 missed cleavages. We set the false discovery rate (FDR) for peptides and proteins at 1%. Proteins labeled as contaminants by the MaxQuant contaminants database were filtered out. The normalized heavy/light ratio was inverted to represent the sample/control ratio and converted to log2 values.

#### Bioinformatics analysis

The Maxquant proteinGroups output file was used for the next steps of bioinformatic analysis. We used the package Proteus (GitHub) that used limma for statistical analysis in R [22] for the differential expression analysis between DEK/NUP214 and each of its mutants, and applied the following filters: a) interacting proteins with an average ratio DEK/NUP214 (or mutant) vs. control ≥ 1.5; b) proteins identified in at least two experiments; c) calculated p.value ≤ 0.05. We cleaned the Protein file data by excluding the IgG proteins as contaminants. For proteins with multiple accession numbers coding for isoforms of the same protein, the first position was kept for further analysis. Proteins identified as DEK and NUP214 were retained in the analysis. The generated output files were used for interaction and pathway enrichment analysis.

#### The molecular interaction search tool (MIST)

MIST database and search tool identifies interacting partners and visualizes protein networks [23]. It is based on curated biological interaction data for significant model organisms and integrated from major public resources such as BioGrid, IntAct, DIP (Database of Interacting Proteins), and mentha. For all MIST analyses, only high-confidence interactions were considered.

#### Search tool for the retrieval of interacting genes/proteins (STRING)

STRING aims to integrate all known and predicted associations between proteins, including physical interactions and functional associations [24].

#### Ingenuity pathway analysis (IPA), interaction network analysis

IPA generates networks from an uploaded data set ranked by score. The score is based on a p-value calculation by the right-tailed Fisher’s exact test and represents a measure of the likelihood that molecules are part of a network by random chance alone.

#### Pathway enrichment analysis

To identify the biological pathways or processes enriched in our analyses, we used g:Profiler and Gene Set Enrichment Analysis (GSEA), combined with the Cytoscape EnrichmentMap application, to visualize the enriched pathways.

**g:Profiler** maps a list of genes to known functional data resources and detects statistically enriched biological pathways or processes [25]. It uses the Ensemble database as the primary resource of information about genes, identifier types, GO (Gene Ontology) terms, and associations. Other data resources are KEGG, REACTOME, CORUM, and human disease phenotype associations from Human Phenotype Ontology and several others [25]. The functional enrichment of a pathway or biological process is statistically evaluated using the cumulative hypergeometric test, and multiple testing correction is performed [25]. The input gene lists used in this study included the interaction partners of DEK/NUP214 and its mutants as separate gene lists, ordered according to the calculated average interaction ratio of ≥ 1.5 over control and a p-value ≤ 0.05.

#### Gene set enrichment analysis (GSEA)

It evaluates the input of biological data at the gene sets or pathways level based on published information about biochemical pathways or co-expression experiments. GSEA determines whether a particular gene set is correlated with one of the two biological states or phenotypes under evaluation (sample classes). For this, an enrichment score (ES) is calculated for each identified pathway and normalized relative to pathway size, resulting in a normalized enrichment score (NES). In this study, the two groups or phenotypes were: 1) unmutated DEK/NUP214; 2) mutated DEK/NUP214. Gene symbol and average log2-fold ratio data were sorted in descending order to create the ranking list. The collection of gene sets used in the GSEA analysis was downloaded from http://baderlab.org/GeneSets as a GMT file containing pathways downloaded from several resources such as GO, REACTOME, CORUM, and others [26].

### Enrichment and in-gel detection of phosphorylated proteins

See S1 Data

### Antibodies

For the antibodies used in this study, see S1 Data.

### Chromatin immunoprecipitation

Cell fractionation was performed using the Nuclear/Cytosol Fractionation Kit (BioVision, Milpitas, CA, USA) according to the manufacturer’s instructions. 48h after transfection, 293T cells were incubated for 15min in hypotonic buffer followed by multiple passages through a 29G cannula for breaking the cytoplasmic membranes. The addition of MgCl2 maintained the nuclear pore complexes [27]. After controlling the integrity of the nuclei, the suspension was centrifuged (5min, 670xg, 4°C). The cytoplasmic proteins were recovered from the supernatant, and the nuclear membrane was lysed by 0.5% NP-40 on ice. Centrifugation separated the nucleosol with the soluble protein fraction from the chromatin. The proteins were precipitated by methanol-chloroform precipitation [27].

### Immunoprecipitation

For (co-)immunoprecipitation, 293T cells were transfected with 5μg of pCDNA3-HA-DEK and pCDNA3-HA-DEK/NUP214 by calcium phosphate precipitation. The transfected 293T cells were harvested in RIPA buffer (50 mM Tris-HCl, pH7.6; 150 mM NaCl; 1% NP-40; 0.5% Na-deoxycholate; 0.1% SDS; 2mM EDTA). The cell suspension was briefly sonicated and the lysates were clarified by centrifugation. Preclearing was performed by incubation for 1h with protein A-Sepharose (Amersham Pharmacia Biotechnology). For the anti-HA-immunoprecipitation, agarose beads covalently coupled to the rat monoclonal anti-HA antibody (Roche—clone 3F10) were used. The beads were washed in RIPA buffer (150 mM NaCl) and resuspended in SDS sample buffer. The immunoblots were stained with the appropriate antibodies.

### Immunoblotting

Immunoblot analyzes were performed according to widely established protocols. Blocking and antibody incubation were performed in 5% low-fat dry milk (Carl Roth). Washing in Tris-buffered saline containing (TBS)(10 mmol/L Tris-HCl pH 8, 150 mmol/L NaCl) containing 0.1% Tween20 (TBS-T) was followed by incubation with a secondary antibody coupled with horseradish-peroxidase for staining with enhanced chemo-luminescence substrate. Blots were “stripped” using “RestoreWestern Blot Stripping Buffer” (Perbio Science). Imaging and elaboration were performed with the LI-COR Odyssey Fc system (LI-COR Biosciences).

### Confocal laser scan microscopy (CLSM)

Cells were cultured overnight on poly-D lysine-covered chamber slides (Corning), washed with TBS, fixed in 4% paraformaldehyde (AppliChem) for 15 minutes, and permeabilized with 0.1% Triton-X in TBS. After blocking in 3% (w/v) BSA (Sigma) and 0.1% Tween20 in TBS for 1h cells were incubated with primary Abs. After extensive washing in TBS-T, we stained the cells with Alexa Fluor 647-conjugated goat anti-rabbit, Alexa Fluor 488-conjugated goat anti-mouse, or Alexa Fluor 647-conjugated mouse anti-rat secondary Abs (Life Technologies). Nuclei were stained with DAPI (Life Technologies). The slides were mounted with Moviol (Sigma). Images were acquired by a Leica TCS-SP5 confocal microscope (Leica Microsystems) under identical conditions for pinhole opening, laser power, photomultiplier tension, and layer number. During data elaboration by Imaris (Switzerland), identical parameters were applied to all samples.

### Gene editing by CRISPR-CAS9

Lentiviral vectors expressing single guide RNAs (sgRNA) targeting exon 3 or exon 5 of CLU were used for genome editing. As a control, we used a lentiviral vector expressing unrelated sgRNA. For editing exon 3, we created pLentiCRISPRv2_CLUh-e3_GGAAGTAAGTACGTCAATA_CAS9 and for editing exon 5 pLentiCRISPRv2_CLUh-e5_CACGAGGCTCAGCAGGCCA–CAS9 [28].

### Quantitative real-time PCR (Q-PCR)

The analysis of HOTAIRM1 expression was performed in FKH-1 cells, U937 cells expressing DEK/NUP214, and 293T DEK/NUP214 expressing cells. Total RNA was isolated using the miRNeasy Kit (Qiagen), and cDNA was synthesized from 20ng of total RNA using the Verso cDNA Synthesis Kit (Life Technologies, UK), according to the manufacturer’s instructions. HOTAIRM1 and the internal control GUSB were amplified using TaqMan Gene expression assays for HOTAIRM1 (Hs03296533_g1) and GUSB (Hs00939627_m1)(Applied Biosystems) as described elsewhere [29].

For miRNA (miR) amplification, the cDNA was obtained using the TaqMan Advanced miRNA cDNA Synthesis Kit (Applied Biosystems) followed by amplification of miR-196a-5p (478230_mir), miR-196b-5p (478585_mir), let-7a-5p (478575_mir), let-7e-5p (478579_mir), miR-644a (479124_mir)(Thermo Fisher Scientific). The miR expression was normalized using a non-human miR159a as spike-in control (Thermo-Fisher Scientific—10620310) added to the RNA sample (1pg in 100ng RNA). After the cDNA synthesis, the spike-in control was amplified using ath-miR159a TaqMan Advanced miRNA Assay (478411_mir, Thermo Fisher Scientific).

All Q-PCR experiments were performed in triplicate, and at least two biological replicates were performed on the QuantStudio5 Real-Time PCR System (Thermo Fisher Scientific). The calculated difference between means corresponded to the ΔΔCt.

The statistical analysis of Q-PCR data was performed in GraphPad Prism 9.0 software (GraphPad, CA, USA) on the ΔCt values for each of the replicate using the Welch’s t-test and a significance level of p<0.05 [30].

## Results

### The expression of DEK/NUP214 disrupts the localization of NUP214 and DEK

**Fig 1:**
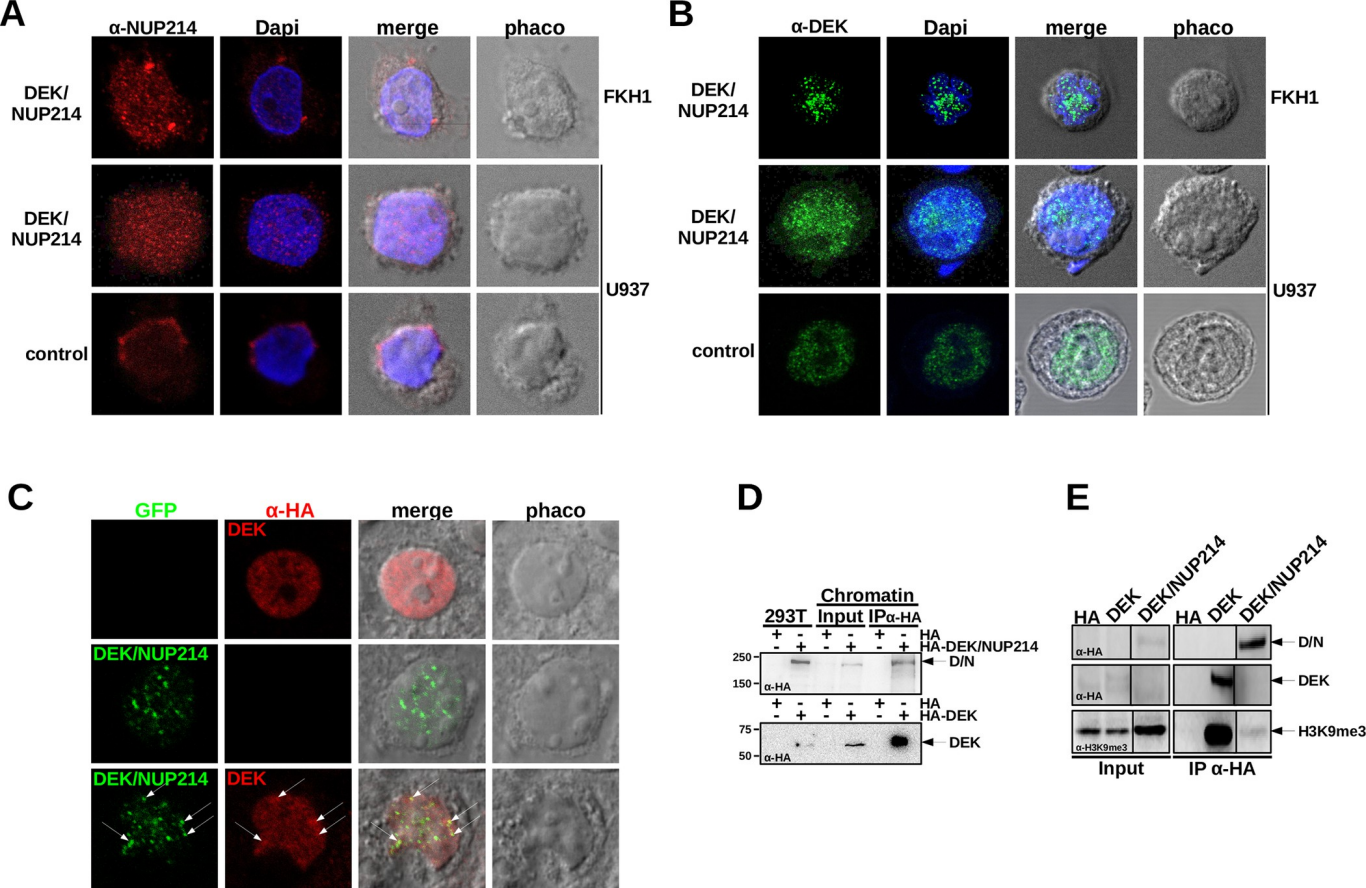
Localization of NUP214, DEK, and DEK/NUP214. **A.** Localization of NUP214 in t(6;9)-AML cells FKH1 and U937 cells stably expressing DEK/NUP214 and U937 control cells—CLSM: red fluorochrome–Alexa 594-conjugated secondary Ab detecting the anti-NUP214 Ab; Dapi–Hoechst 3334 staining; phaco–phase contrast; merge–digital superposition. **B.** Localization of DEK and DEK/NUP214 in t(6;9)-AML cells FKH1 and U937 cells stably expressing DEK/NUP214 and U937 control cells–CLSM: green fluorochrome—Alexa 488-conjugated secondary Ab detecting the anti-DEK Ab. Dapi–Hoechst 3334 staining; phaco–phase contrast; merge–digital superposition. **C.** colocalization of DEK and DEK/NUP214 in transfected 293T cells; red fluorochrome–Alexa 594-conjugated secondary Ab detecting the anti-HA Ab detecting HA-tagged DEK; green fluorochrome–GFP signal of the GFP-tagged DEK/NUP214; phaco–phase contrast; merge–digital superposition. **D.** Chromatin-IP of DEK and DEK/NUP214. HA-tagged DEK and DEK/NUP214 transfected into 293T cells. Immunoblot anti-HA, Input– 10% of chromatin/protein lysate used for the IP. E. Co-IP anti HA between HA-tagged DEK or DEK/NUP214 and intrinsic H3K9me3 in 293T cells. Input– 10% of the cell lysate used for the Co-IP. The immunoblot was stained either with the anti-HA Ab (for detecting DEK and DEK/NUP214) or the anti- H3K9me3 Ab.

A leukemogenic translocation frequently leads to the disruption of intracellular structures, such as nuclear bodies, by mislocalization and an altered functionality of interaction partners [3]. Therefore, we assessed the influence of DEK/NUP214 on the localization of normal DEK and normal NUP214 by confocal laser scan microscopy (CLSM). First, we investigated the localization of NUP214 in t(6;9)-positive FKH1 by using an anti-NUP214 antibody (red fluorochrome) to detect both NUP214 and DEK/NUP214. DAPI confirmed the integrity of the nucleus. In FKH1, the anti-NUP214 signal exhibited two patterns: perinuclear dots, representing the physiological localization of NUP214 in the nuclear pore complex, and microspeckles in the nucleus related to DEK/NUP214 itself or NUP214 bound to DEK/NUP214 (Fig 1A).

Next, we studied the effect of DEK/NUP214 on normal NUP214 localization in cells with two normal alleles of NUP214. Here, we compared U937 cells transfected with an empty vector and U937 cells stably expressing DEK/NUP214 [18]. In contrast to the physiological perinuclear pattern of NUP214 in U937 control cells (Fig 1A), the anti-NUP214 signal in DEK/NUP214 expressing cells exhibited a microspeckled nuclear pattern (Fig 1A), indicating the ability of DEK/NUP214 to disrupt the physiological localization of NUP214.

Next, we investigated the localization of DEK in FKH1 cells (green fluorochrome). The anti-DEK signal exhibited a unique microspeckled pattern (Fig 1B) similar to the anti-NUP214 Ab. Similarly, DEK/NUP214 expressing U937 cells exhibited a microspeckled anti-DEK signal (Fig 1B) comparable to the anti-NUP214 signal (Fig 1A) but indistinguishable from that of endogenous DEK in U937 control cells (Fig 1B).

To answer whether DEK and DEK/NUP214 co-localize and influence each other, we co-expressed an HA-tagged DEK with a GFP-tagged DEK/NUP214 in 293T cells. The anti-HA signal of DEK (red fluorochrome) showed a microspeckled pattern apparently different from that of the DEK/NUP214 associated GFP signal (green fluorochrome). However, it should be noted that the HA signal comprises the specific labeling of HA-DEK and background signaling resulting from unspecific antibody binding. In cotransfected cells, the electronic superposition of the red (DEK) and green (DEK/NUP214) signals revealed a partial colocalization of DEK and DEK/NUP214 (indicated by white arrows), whereby not all green signals corresponded to red signals and vice versa and only in few cases there was a complete overlap of the red and the green fluorochrome (Fig 1C). Therefore, we considered DEK and DEK/NUP214 partially co-localizing.

The partial colocalization of DEK and DEK/NUP214 prompted us to investigate whether the fusion of DEK to NUP214 would change DEK/NUP214’s association with chromatin-mediated by the DEK portion [27]. Therefore, we localized DEK and DEK/NUP214 in the chromatin of 293T cells transfected with HA-tagged DEK or DEK/NUP214 by an anti-HA chromatin-immunoprecipitation. Although we extracted both from chromatin, only DEK interacted with H3K9me3 but not DEK/NUP214 (Fig 1E).

These data provide evidence that the t(6;9) disrupted the perinuclear localization of NUP214 in the nuclear pore complex. On the other hand, DEK/NUP214 exhibited a microspeckled localization that partly overlapped with that of DEK. In addition, the DEK portion of the fusion protein loses at least some DEK interactions within the chromatin.

### The DEK/NUP214 interactome determined by tandem affinity proteomics (TAP)

Based on our findings, we considered three scenarios: i.) interactors of DEK and NUP214 exhibit either a loss of function or a modified function in the context of the t(6;9) fusion; ii.) novel interactors appear that would not interact with wt DEK or wt NUP214; iii.) interactors of DEK or NUP214 do not bind to DEK/NUP214.

**Fig 2:**
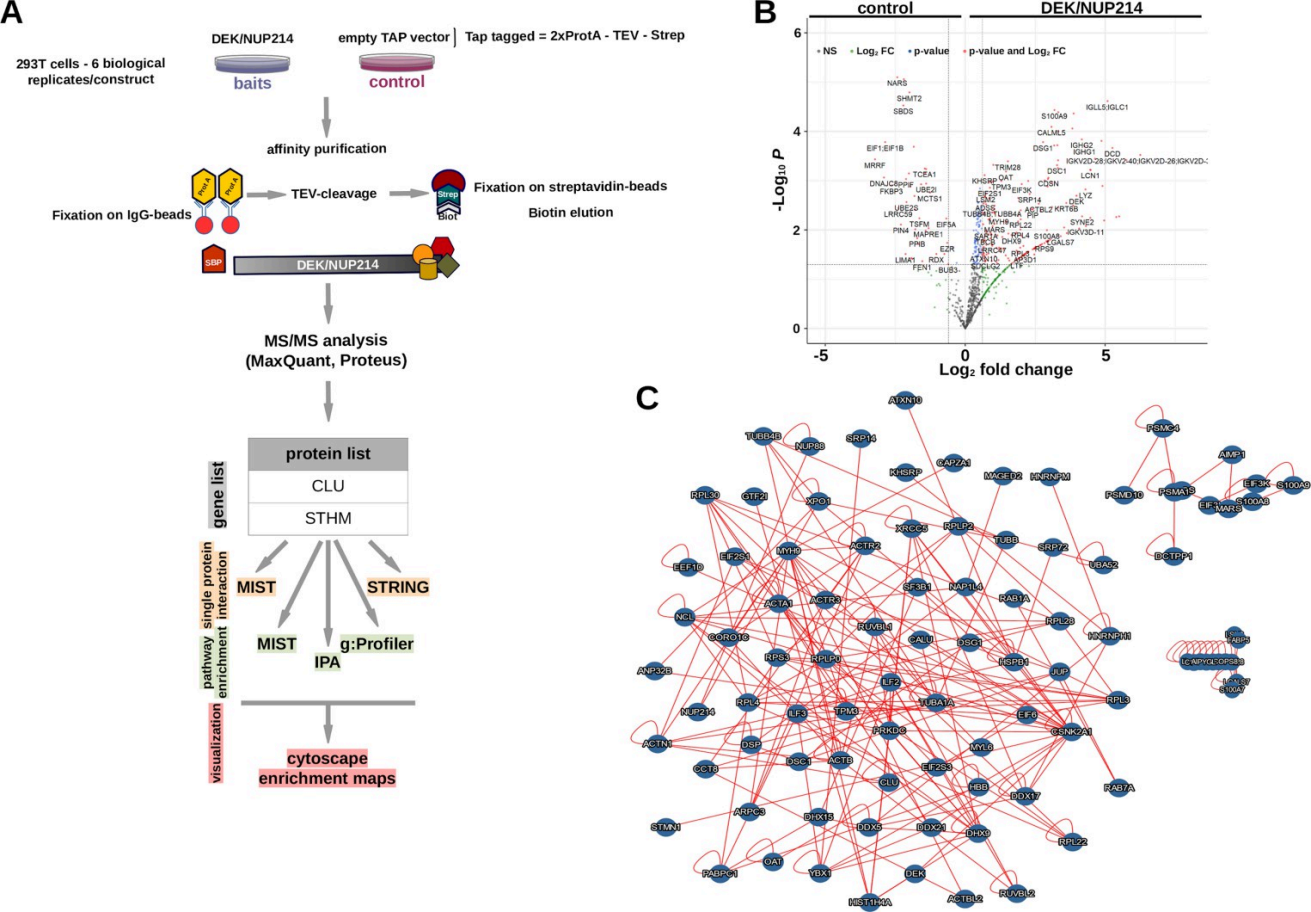
TAP-tag proteomics. **A.** TAP—Tandem Affinity Purification; TEV–Tobacco Etch Virus protease; Prot A–protein A; SBP–Streptavidin Binding Protein. Workflow of TAP-tag proteomics and Tandem MS followed by data elaboration (MaxQuant, Proteus) in combination with extended data mining by MIST, STRING, IPA, and g:Profiler. g:Profiler results were further refined and graphically elaborated in Cytoscape using the EnrichmentMap application. **B.** Volcano blot (Proteus) was created to distinguish DEK/NUP214 and empty vector interactomics—a synopsis of potential DEK/NUP214 interactors. **C.** MIST analysis of DEK/NUP214 interactors applying a threshold of Log2 fold change corresponding to an increase of interaction of 1.5-fold and a p<0.05.

All three scenarios are closely linked with the protein-protein interaction of DEK/NUP214. Therefore, we investigated the interactome of DEK/NUP214 by TAP-tag proteomics. First, we fused DEK/NUP214 to a tag composed of two Protein A sequences separated from a Streptavidin binding sequence by a TEV recognition site (ENLYFQ)(Fig 2A) [31]. Then, we controlled the biological functionality of this construct as a leukemogenic oncoprotein in a CFU-S12 assay (S1A Fig).

To quantify our findings and further improve specificity, we combined TAP-purification with SILAC labeling (see Material and Methods) [32]. We created Svy cells that were then transfected with the empty vector (TAP) as a reference and control, whereas the Sli cells were cultured in an unmodified medium and transfected with the TAP-DEK/NUP214 construct. We schematically show the entire workflow in Fig 2A. We performed six biological replicates and applied the criteria for defining a candidate protein: i.) an average ratio ≥ 1.5 versus control; ii.) differences with a p-value ≤ 0.05; and iii.) identification in ≥ 2 replicates.

The volcano plot showed the proteins precipitated preferentially and significantly by DEK/NUP214 compared to the empty vector control (Fig 2B; see also S1 Table). It included several proteins of the Ig heavy and light chain family that we eliminated from further analysis as false interactors. Among the so-defined candidate proteins, we found NUP88 and XPO1, both already known as interaction partners of DEK/NUP214 [33,34,35], confirming the validity of our investigation. Although DEK/NUP214 is a nuclear protein, NUP214 is a member of the nuclear pore complex involved in the nuclear-cytoplasmic transport of RNA and proteins [34] at the outer side of the nuclear membrane making its interaction with cytoplasmic proteins as likely as with nuclear proteins. Only 56% (72/130) of proteins interacting with DEK/NUP214 were nuclear (S2 Table).

The first step of bioinformatic elaboration of the data was the analysis using MIST to identify interactions between the entirety of interactors to DEK/NUP214 (see Material and Methods). This analysis revealed a strong association among the interaction partners of DEK/NUP214 forming one large and two small protein networks (Fig 2C). The next layer of interaction partners of all the interactors and DEK/NUP214 gave an idea about the dimension and complexity of DEK/NUP214’s biological network S2 Fig).

### DEK/NUP214 interacts with members of protein complexes involved in RNA processing, ribosome biogenesis, and cell shape and motility

**Fig 3:**
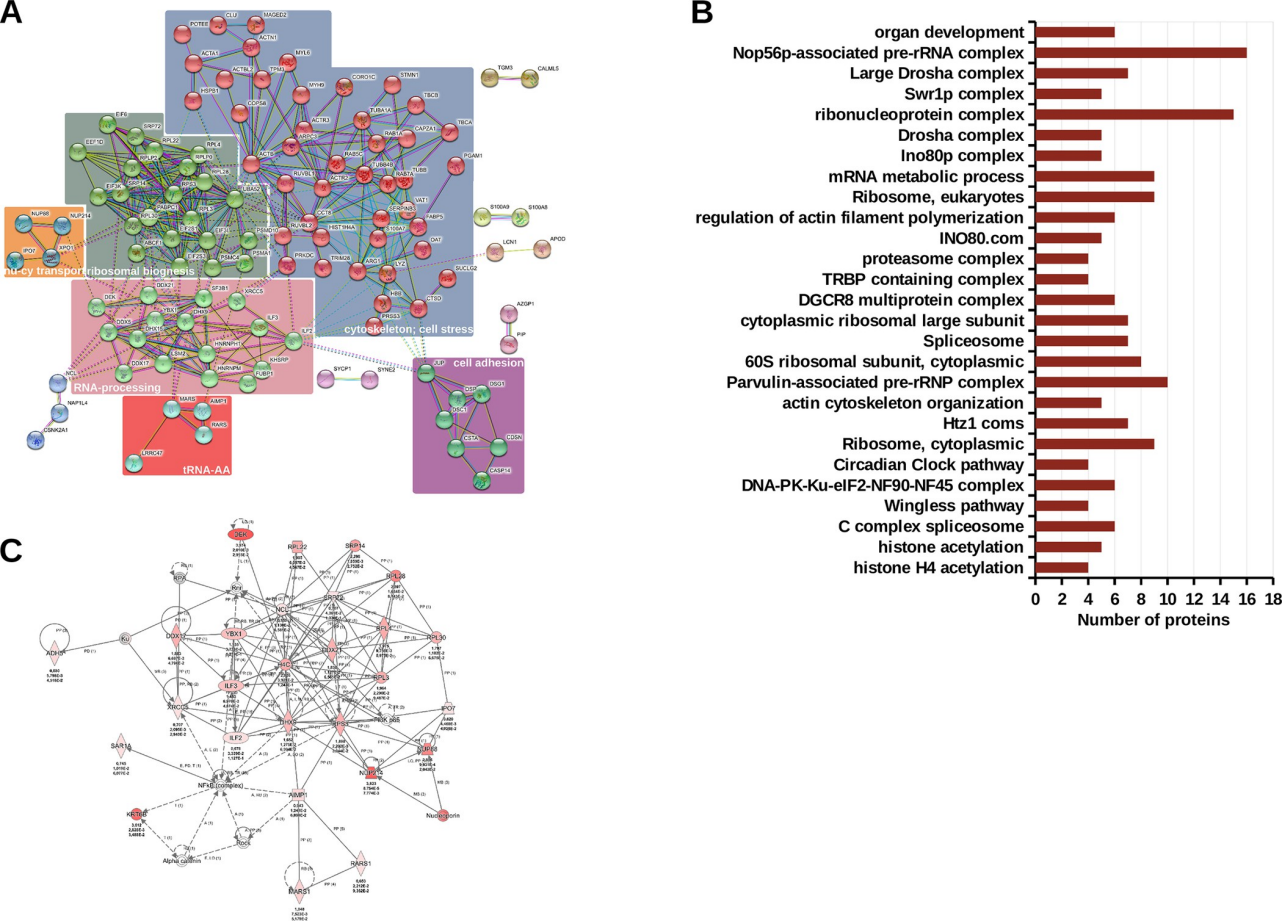
Functional analysis of DEK/NUP214 interacting complexes. **A.** STRING analysis. The STRING algorithm identified 6 clusters of at least four interactors indicating their function as evidenced by different colors. **B.** MIST analysis of the protein complexes formed by DEK/NUP214 interactors and their function–threshold was set at a minimum of 4 interactors. **C.** IPA network analysis of DEK/NUP214 interactors shows the network with the lowest p-value, the "RNA damage and repair network," corresponding to 27 DEK/NUP214 interactors involved in this single network. Each node in the network represents a gene, and the solid or dotted lines connecting them show a direct or indirect association, respectively. Red/grey nodes were the input genes detected in this study; white nodes are molecules inserted by IPA from the Ingenuity Knowledge Base.

To organize this complex network of DEK/NUP214’s interactome, we first investigated the enrichment of potential functions and functional categories by STRING [24]. The parameters for the analysis were set at high confidence and limited to the first shell of interaction, meaning that we considered only the direct interaction partners of DEK/NUP214. Here we show the analysis using the cluster tool of STRING. >4 interacting proteins defined a cluster. STRING evidenced 6 clusters that predicted an active involvement of DEK/NUP214 in several processes through its interaction partners. One cluster was nuclear-cytoplasmic transport, two were related to the cytoskeleton and cell adhesion, respectively, and three pointed to RNA-processing and ribosomal biogenesis (Fig 3A).

In contrast to STRING, MIST creates evidence-based networks based on ranked interactions (see Material and Methods). As suggested by S2 Fig, each interaction between DEK/NUP214 and a given protein would finally be an interaction between DEK/NUP214 and the complex this protein is part of. Thus, we analyzed DEK/NUP214 interactome by MIST filtering for only highly ranked interactions [23]. In this way, we identified about 130 complexes, most related to RNA-processing, splicing, generation of miR, and ribosome biogenesis but also with transcription regulation by epigenetic mechanisms such as acetylation and chromatin modeling (S2 Table). Fig 3B shows all complexes involving ≥4 DEK/NUP214 interactors, most of them involved in RNA processing and metabolism.

To improve the biological interpretation of the MIST output, we performed a Network analysis of the DEK/NUP214 interactome in IPA (see Material and Methods). We revealed the cooperation among groups of proteins by identifying direct or indirect connections between them and relating them to known diseases and biological functions. The highest score and thus the lowest p obtained in the network analysis of the DEK/NUP214 interactome was 56 for the RNA damage and repair network corresponding to 27 DEK/NUP214 interactors involved in this single network (Fig 3C).

### DEK/NUP214 is involved in processes related to RNA splicing and stability, regulation of gene expression, and immunity

To better understand the connection between the DEK/NUP214 interactome and disease mechanisms, we performed a pathway enrichment analysis by g:Profiler that condensed the list of interactors into a list of interpretable pathways [25](see Material and Methods). Thus, we identified related pathways and major biological themes. The visualization and interpretation of pathway enrichment analyses were performed in Cytoscape using the EnrichmentMap and AutoAnnotate tools as described [36].

**Fig 4:**
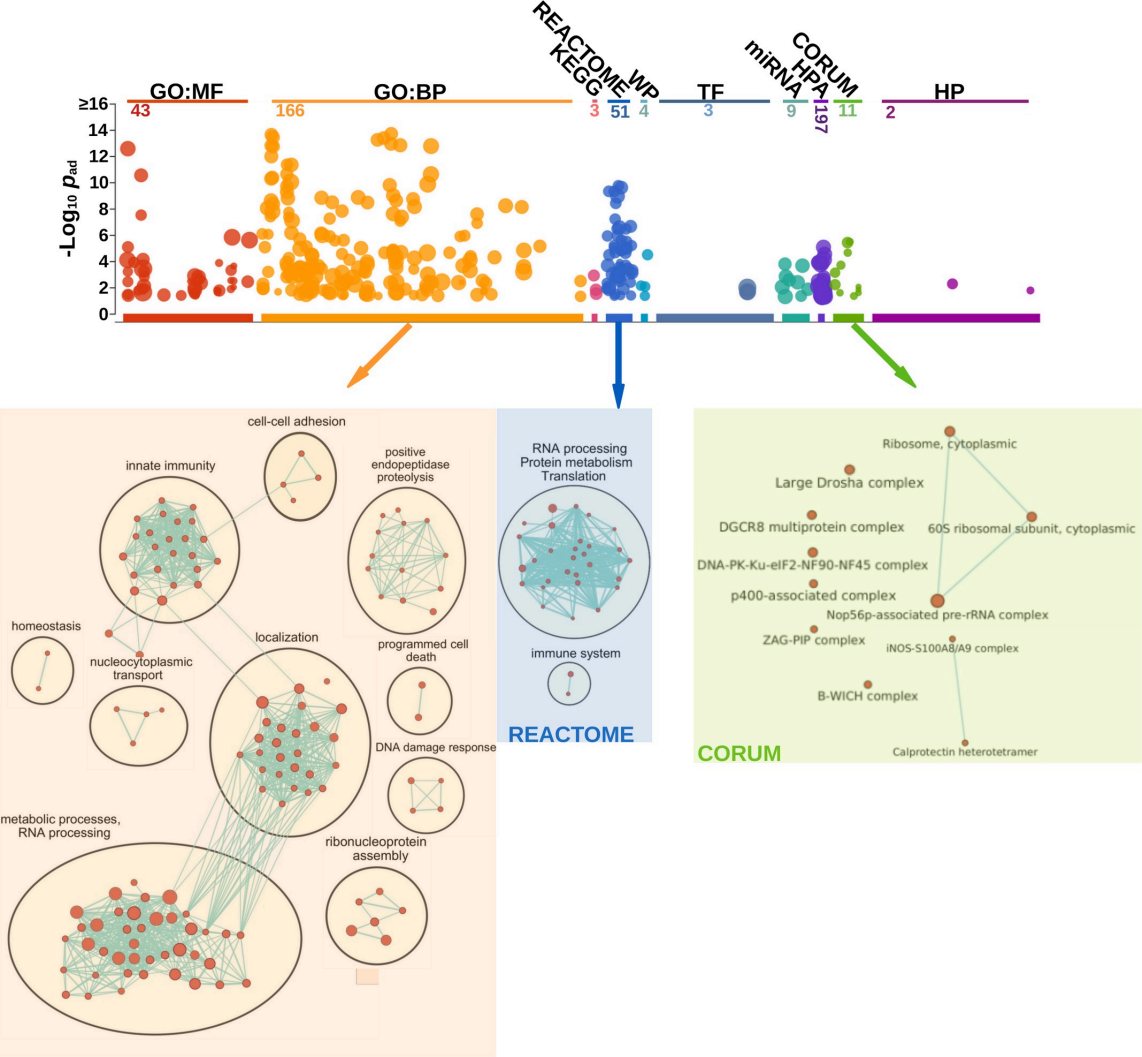
Bioinformatic analysis of biological processes of DEK/NUP214 interactors in g:Profiler and Cytoscape. g:Profiler–Shown are the numbers of the statistically relevant (p≤0.05) processes in the different resources of g:Profiler (GO: MF, GO: BP, KEGG, REACTOME, WP, TF, miR, HPA, CORUM, and HP). Here we show the refinement of the g:Profiler output from GO: BP, REACTOME, and CORUM by Cytoscape/EnrichmentMAP. As evidenced by connecting lines, the edge cut-off for protein overlap between processes was set at 0.7 for GO: BP and 0.5 for CORUM and REACTOME. All sets had a p≤0.05.

First, we ordered the proteins according to the interaction ratio between DEK/NUP214 (Sli) and empty vector control (Svy). Next, we selected processes only with an FDR of ≤0.05 and an adjusted p≤0.05 (Fig 4). The GeneOntology (GO:BP) analysis gave four major biological processes: a. RNA and protein metabolism (RNA splicing and stability, regulation of gene expression), b. intracellular localization (transport of macromolecules, protein targeting to ER, localization to membrane), c. innate immunity (leukocyte activation, leukocyte degranulation), and d. regulation of proteolysis.

The most prominent group was "metabolic processes and RNA-processing." With its 38 interactor proteins, this node exhibits multiple functions in the cell, including transcription regulation by binding to histones, RNA polymerase, and DNA, and by helicase activity. Two nodes in this group represented protein metabolic processes. One can find details about this and all the other nodes in the S3 Table. The multiple lines indicated the interconnection between this group of processes and the one of "localization" due to multiple proteins involved in both processes. Hence, many different nodes shared proteins broadly related to transcription/translation regulation; therefore, we related them to gene expression regulation. In addition, DEK/NUP214 was involved in biological processes, including ribonucleoprotein complex assembly, cell death, and response to DNA damage. "Localization" and "Innate Immunity" were probably related to the fact that DEK/NUP214 is active in a myeloid context and linked to the intracellular network of structural proteins such as tubulins and actins. We revealed that several DEK/NUP214 interactors were involved in multiple processes, including DDX5, DDX21, DHX9, YBX1, HNRNPM, SF3B1, and RPS3 (S3 Table).

We evidenced two complexes using REACTOME as a resource: i.) RNA-processing, protein metabolism, and translation, and ii.) innate immunity.

CORUM is a resource that provides protein complexes as output. It revealed closely related RNA processing complexes, such as the Large Drosha complex, the DGCR8 multiprotein complex, and the B-WICH complex, in addition to the ribosomal complexes interconnected with the NOP56p associated pre rRNA complex. Drosha and DGCR8 are involved in miR precursor processing and synthesis [37]. All these findings indicate a significant role of DEK/NUP214 in RNA-processing from ribosomal biogenesis, RNA splicing, and stability to miR synthesis.

### Reduction of complexity: the interactome of three biologically inactive mutants of DEK/NUP214

The complexity of the protein networks and the related signaling pathways affected by DEK/NUP214 prompted us to investigate which interactors are indispensable for the leukemic phenotype induced by this oncoprotein. Therefore, we analyzed the interactomes of three biologically dead mutants of DEK/NUP214 and compared them with that of unmutated DEK/NUP214 (see S1 Data and S3 Fig). We hypothesized that most interactors would be shared. However, those most relevant would either not bind or strongly reduce their interaction with the mutants compared to the unmutated DEK/NUP214. We used two types of mutants: i.) phosphorylation mutants in the DEK portion of the fusion protein where point-mutations eliminated putative GSK3 phosphorylation sites (ΔP1/NUP214) followed by the deletion of putative CKII phosphorylation sites (ΔP2/NUP214) [38,39]; ii.) coiled-coil (CC) mutants in the NUP214 portion defined by the I-Tasser analysis (S3A–S3E Fig). Our biological analysis of the mutants revealed that intact phosphorylation sites and the presence of helix 3 were indispensable for the leukemogenic potential of DEK/NUP214 (S3F Fig). CLSM and nuclear fractionation analyses excluded a change in localization as a cause for the loss of leukemogenic potential of the DEK/NUP214 mutants. All mutants co-localized with unmutated DEK/NUP214 (S4A and S4B Fig).

**Fig 5:**
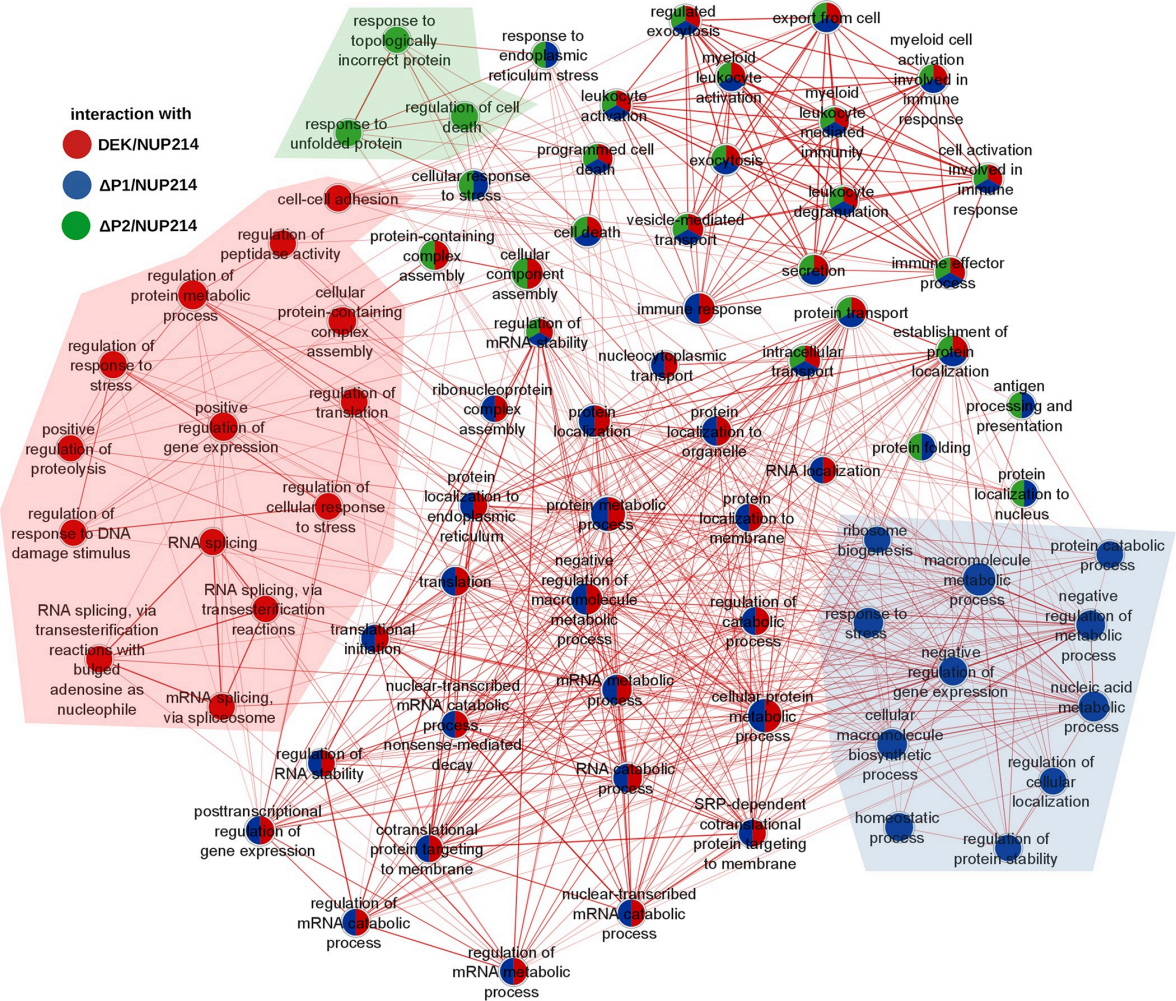
Bioinformatic analysis of biological processes comparing the interactome of DEK/NUP214 with those of its mutants in g:Profiler and Cytoscape. Gene set size was set as 10–100 genes/set. The output from the analysis of the interactomes in g:Profiler was refined and compared in Cytoscape/EnrichmentMap using an edge cut-off of 0.7. A set had a p≤0.05). Red: DEK/NUP214; blue: ΔP1/NUP214; green: ΔP2/NUP214.

Based on the assumption that comparing the interactome of DEK/NUP214 with that of its mutants should allow us to evidence the interactors and their related signaling pathways indispensable for the leukemogenic potential of DEK/NUP214, we performed an unsupervised investigation using g:Profiler. DEK/NUP214-interactors were involved in pathways related to RNA splicing, cell stress, cell adhesion, proteolysis, and DNA damage. In total, 14/78 processes involved exclusive interactors of DEK/NUP214 (Figs 5 and S5 and S4 Table).

**Fig 6:**
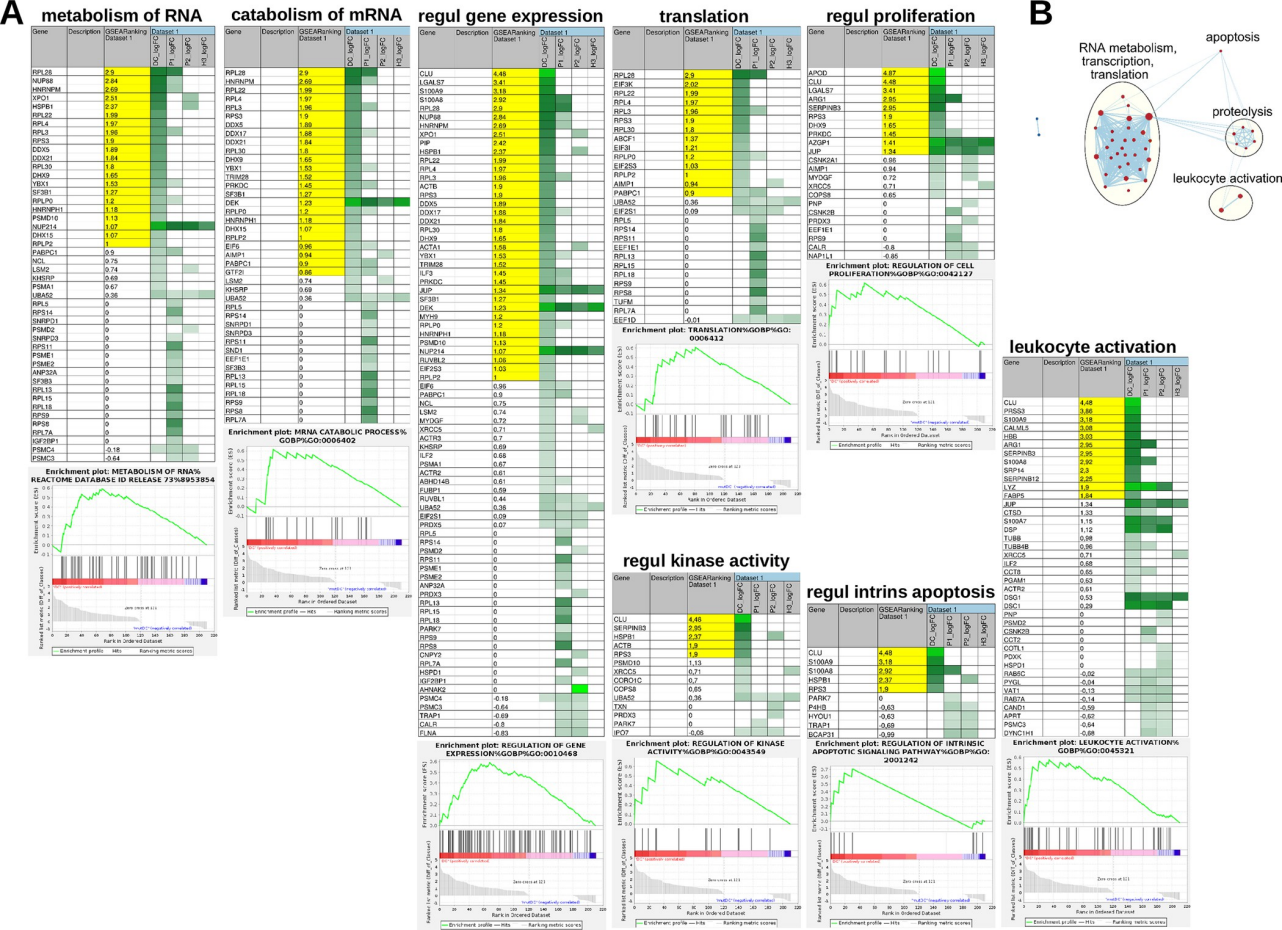
Bioinformatic analysis of biological processes comparing the interactome of DEK/NUP214 with those of its mutants by GSEA and Cytoscape–quantification of interactions. The filter in GSEA was set at a minimum of 10 genes/set. **A.** Shown are the eight most statistically significant processes revealed by GSEA. DC_logFC, P1_logFC, P2_logFC, and H3_logFC- the data set names given by GSEA to the list of interactors of DEK/NUP214, ΔP1/NUP214, ΔP2/NUP214, and DEK/ΔH3B, respectively. Yellow—leading-edge subset proteins responsible for the enrichment of the respective gene sets. The intensities of green correlate with the strength of interaction between DEK/NUP214, ΔP1/NUP214, ΔP2/NUP214, or DEK/ΔH3B and the given interactors. **B.** The analysis of the interactomes of DEK/NUP214 and its mutants in GSEA was refined in Cytoscape/EnrichmentMap, showing the processes highly enriched in the interactome of DEK/NUP214.

Although DEK/NUP214 and its biologically dead mutants shared other RNA-associated pathways, such as ribonucleoprotein assembly, regulation of RNA stability, and RNA metabolic processes (primarily by ΔP1/NUP214)(Fig 5), this type of analysis did not take into account the strength of interaction. Thus, it does not differentiate between strong and weak protein-protein interactions. Therefore, we performed a supervised comparison of our data sets including the quantification of the interactions. First, we created a ranked list of genes in GSEA from the two sample classes: class I—DEK/NUP214; class II—the respective DEK/NUP214 mutant, with the ranking based on the strength of interaction of each protein in each of the samples (see Material and Methods, S5 Table). An ES reflects how a gene set correlated with one of the two sample classes. As not all the members of the gene set will equally participate in a given biological process, extracting the core members that account for the gene set’s NES helps to identify biologically relevant genes, the so-called leading-edge (Fig 6A). Thereby, we identifiedFig 4 groups of independent processes (at a p-value ≤0.05, FDR value ≤0.35) that we considered directly involved in the determination of the DEK/NUP214-related phenotype (Fig 6B). The most prominent group includes the processing of RNA and all directly related processes such as regulation of gene expression, translation, and proliferation. Smaller subgroups were proteolysis, apoptosis, and leukocyte activation (Fig 6B). The proteins accounting most for the enrichment of these gene sets in DEK/NUP214 (the leading-edge proteins marked in yellow) were involved in the metabolism of RNA and regulation of gene expression (DDX5, DDX21, RPS3, SF3B1, YBX1, DHX9). In contrast, others like Clusterin (CLU) and S100A9 were highly involved in leukocyte activation, apoptosis regulation, and gene expression (Fig 6A).

### DEK/NUP214 controls the expression of lncRNA HOTAIRM1 and miRs

**Fig 7:**
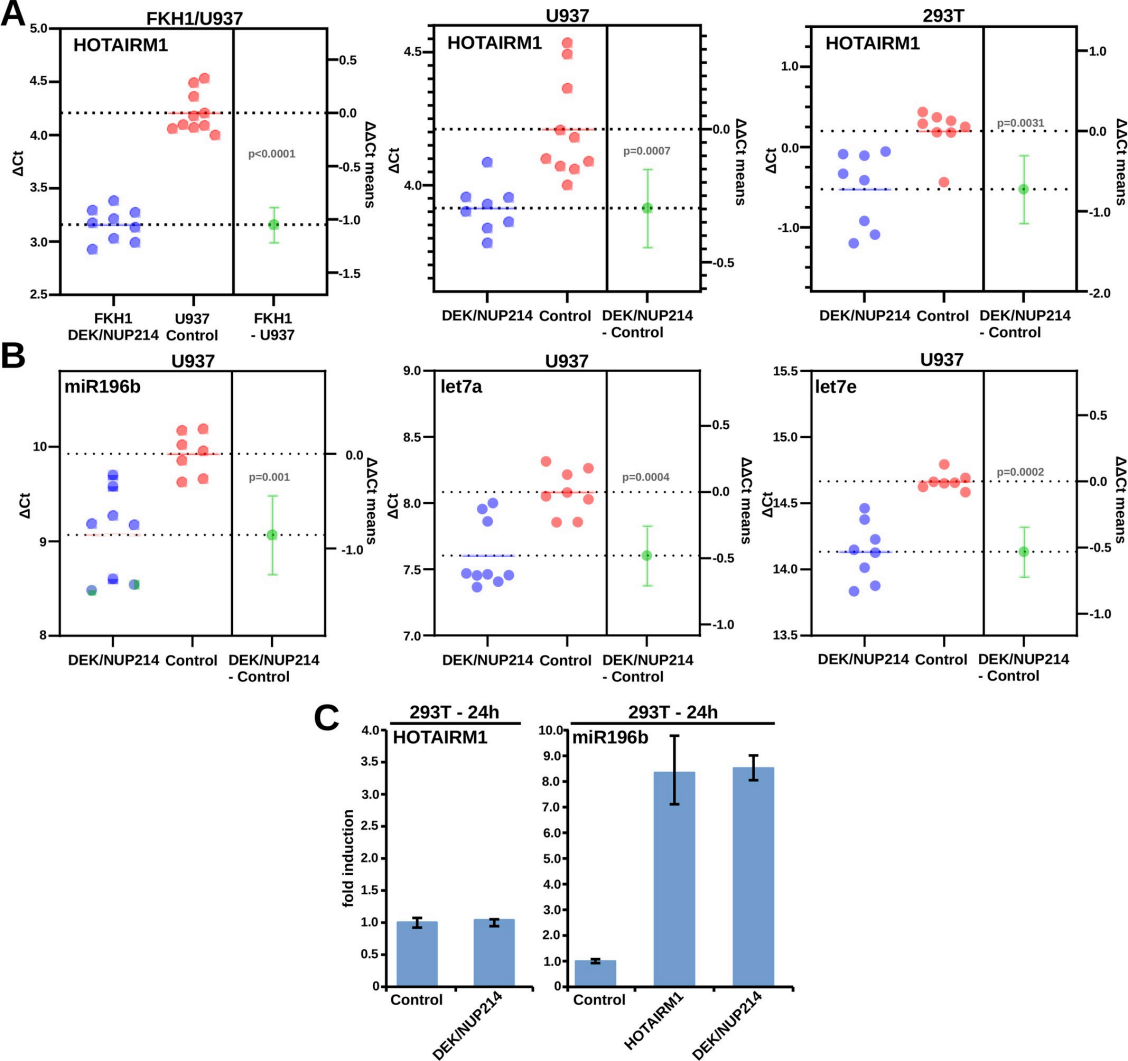
Validation of RNA-related processes and interactions with DEK/NUP214. **A.** Relationship between DEK/NUP214 and HOTAIRM1 expression: Q-PCR: estimation plots comparing ΔCt values and extracting the ΔΔCt for statistical analysis (GraphPad). FKH1 cells: t(6;9)-positive cell line; U937: DEK/NUP214 negative cells; U937-DEK/NUP214: stably expressing DEK/NUP214; 293T: empty vector controls; 293T-DEK/NUP214: 293T cells transiently transfected with DEK/NUP214. **B.** Relationship between DEK/NUP214 and miR196b, let7a, and let7e in U937 cells stably expressing DEK/NUP214. **C.** Relationship between HOTAIRM1 and miR196b in DEK/NUP214 positive cells.

Modifying the metabolism of RNA should result in modifications of RNA species such as lncRNA and sncRNA. t(6;9)-AMLs are characterized by the over-expression of lncRNA HOTAIRM1 and a specific miR pattern. HOTAIRM1 and miR-196b were independent prognostic factors in t(6;9)-AML [29]. Therefore, we wanted to study the relationship between DEK/NUP214 on one side and HOTAIRM1 and miR expression on the other. Taqman Q-PCR assessed HOTAIRM1 and miR expression in DEK/NUP214 positive cells (FKH1, U937-DEK/NUP214, and 293T-DEK/NUP214) and DEK/NUP214 negative control cells (U937 and 293T cells). As shown in Fig 7A, the presence of DEK/NUP214 led to an up-regulation of HOTAIRM1 in FKH1, U937 stably transfected with DEK/NUP214, and 293T cells transiently transfected with DEK/NUP214. DEK/NUP214 seemed to up-regulate the miRs miR-196b, let7a, and let7b (Fig 7B). As both HOTAIRM1 and miR-196b are localized within the HOXA cluster on chromosome 7, we wanted to disclose whether DEK/NUP214 can directly induce the expression of miR-196b or if it needs the up-regulation of HOTAIRM1. As the induction of HOTAIRM1 in 293T cells by DEK/NUP214 is a late event visible 36-48h after transfection, the up-regulation of miR-196b by DEK/NUP214 at 24h was HOTAIRM1-independent (Fig 7C).

Overall, our findings show an aberrant influence of DEK/NUP214 on the expression of lncRNA and miRs, both used for the risk assessment of patients with t(6;9)-AML.

### CLU co-localizes with DEK/NUP214, and its down-regulation induces apoptosis in DEK/NUP214-positive cells

**Fig 8:**
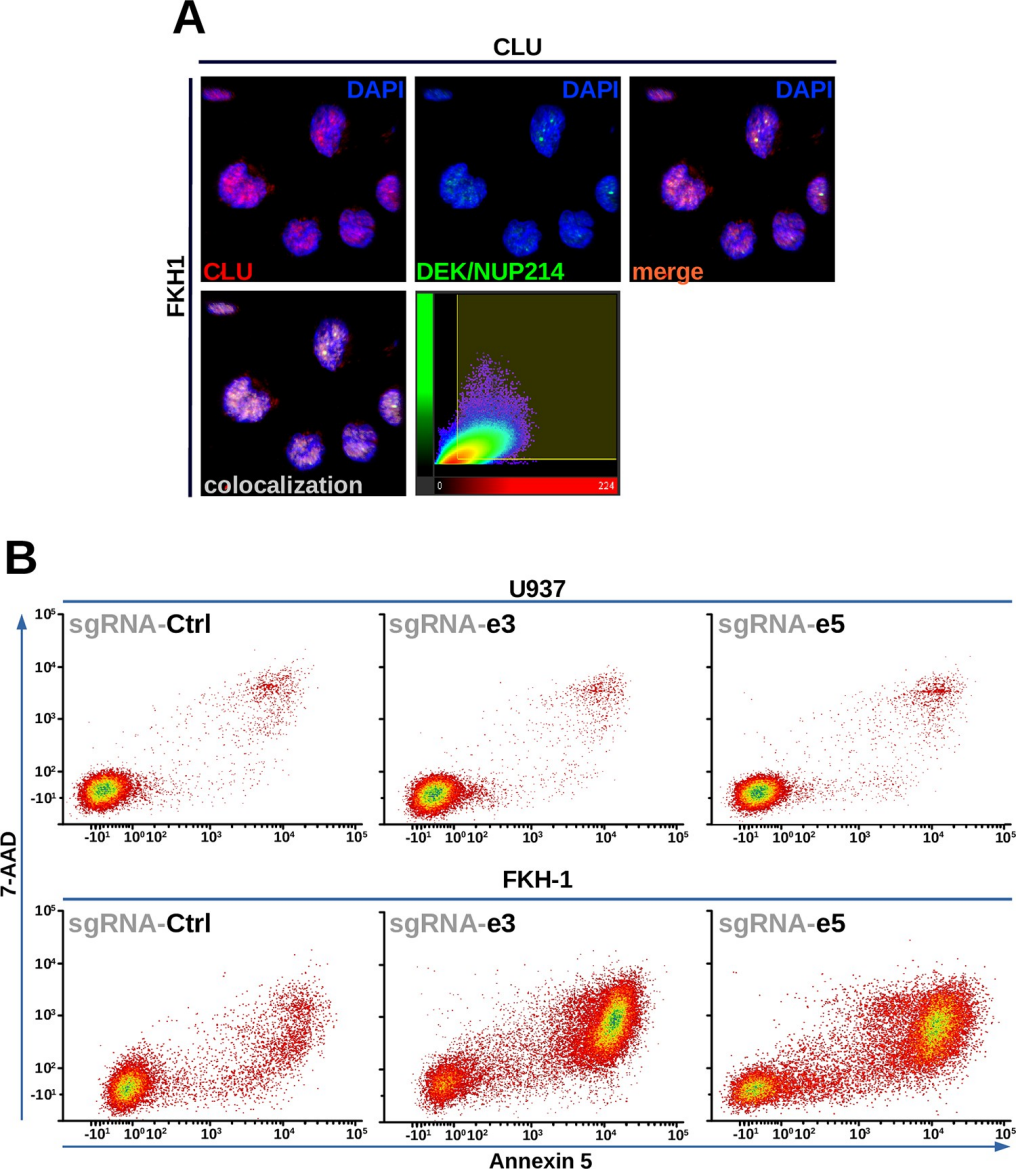
Validation of interactions with DEK/NUP214. **A.** colocalization of CLU and DEK/NUP214 in FKH1 cells: CLSM and analysis of colocalization in Imaris (Switzerland). **B.** Gene editing by CRISPR-CAS9 of CLU in FKH1 cells compared to U937 control cells–apoptosis (7-AAD/Annexin 5). The guidance RNAs were directed against CLU exon 3 (e3) and 5 (e5). Given is one representative experiment of two performed that gave similar results.

As it was highly involved in 5/8 of the identified gene sets showing a high grade of interaction with DEK/NUP214, we took CLU as an example, first for the validation of physical interaction with DEK/NUP214, and second for the validation of the functional relevance of this interaction. CLU is a multi-functional protein, and there are different forms of CLU: one is secreted, and the others are localized either in the cytoplasm or in the nucleus. It is involved in cancer cell proliferation, stemness, survival, therapy resistance, and inhibition of programmed cell death [40]. As TAP is a very restrictive double co-immunoprecipitation, we opted for a colocalization assay to validate the interaction between CLU and DEK/NUP214. Therefore, we assessed colocalization by the data analysis performed by the colocalization tool of the Imaris software (Imaris, Switzerland). As shown in Fig 8A, DEK/NUP214 and CLU co-localized in the nucleus of DEK/NUP214-positive FKH1 cells, and all parameters supported colocalization (S6 Table). In this way, we could exclude that the interaction DEK/NUP214-CLU occurred outside the nucleus.

For functional validation, we targeted CLU by CRISPR-CAS9. All lentivirally expressed gRNAs, directed against exon 3 (gRNA3) or exon 5 (gRNA5), induced apoptosis in the DEK/NUP214-positive FKH1 cells, but not in control cells, where the apoptosis induction was comparable to that induced by the unrelated gRNA used as a negative control.

These results suggest that our subtractive proteomics approach could identify real interactors of DEK/NUP214 with functional relevance for the leukemogenic phenotype.

## Discussion

This study aimed to disclose the role of the localization of DEK/NUP214 and the related sequester of its interacting proteins for the induction of t(6;9)-AML. Here we show the complexity of the biological consequences of the expression of DEK/NUP214.

The DEK portion seems responsible for the DEK/NUP214 localization as DEK is nearly entirely conserved in DEK/NUP214 [35,18,27] and DEK and DEK/NUP214 localize to nuclear speckles in hematopoietic cell models, as others have already shown in non-hematopoietic cell models [34,41,35]. Nevertheless, its fusion to NUP214 disrupted some of the interactions of DEK at the molecular level, and thus the localization of DEK itself and its functionality has to be considered disrupted in DEK/NUP214-positive cells. We also found a strong influence on the localization of NUP214, which seemed to be dependent on the expression level of DEK/NUP214. FKH1 cells express DEK/NUP214 at a physiological level, supposedly not enough to sequester NUP214 entirely. Therefore, an anti-NUP214 signal could still be seen in the nuclear pore complex. In contrast, transfected U937 cells express higher levels of DEK/NUP214 and NUP214 was seen only inside the nucleus.

The pull-down of already known interactors of NUP214, such as CRM1 (exportin1 or XPO-1) and NUP88 [42,34], or of DEK, such as DDX21 [43], served as quality control of the interactomics experiments. It confirmed the underlying idea that mislocalization of DEK or NUP214 either changes the localization of their interactors, creates new interactions, or abolishes their interactions. Such a sequester of interaction partners can also support novel functions due to the novel environment, as already shown for other leukemogenic fusion proteins like PML/RARα, PLZF/RARα, or RUNX1/ETO [10,3,2].

Proteomics and bioinformatics have enormous potential for the disclosure of mechanisms of oncoprotein-mediated leukemogenesis. First, we showed the complexity of the networks DEK/NUP214 was part of by determining the connections among the interactors and the complexes they formed. Here we showed the functionality of the interactors, the signaling pathways they were involved in, and their importance for a given signaling pathway. As a result of this, we revealed the existence of a relationship between the interactome and the DEK/NUP214-mediated leukemogenesis.

Our analyses were complicated by apparently independent but overlapping gene sets (leucocytes-, myeloid cells-, white blood cells-activation) deposited on the gene set databases. Each gene set represents the result of single investigations with slightly different aims and models.

Another critical point of bioinformatics is the complexity of the results or, in other words, the background created by true interactors but probably not relevant for the induction of DEK/NUP214 induced phenotype. We reduced complexity by analyzing the interactomes of three biologically inactive DEK/NUP214-mutants. In addition, the data have to be quantified because an unsupervised analysis with its yes/no results does not pick quantitative differences. These differences can influence functionality, as reported for PU.1, where a slight reduction of its functionality is responsible for the induction of leukemia [44]. Therefore. we used inactive mutants and the quantification by SILAC to separate significant signaling pathways from those that we did not consider relevant for determining he leukemic phenotype mediated by DEK/NUP214.

Our bioinformatic analysis of the DEK/NUP214 interactome revealed a strong interaction with important complexes involved in RNA processing, RNA metabolism, ribosome synthesis, DNA repair mechanisms, and leukocyte activation. RNA processing includes developing the different forms of RNA precursors into more mature forms. The formation of mRNA involves spliceosomes, the synthesis of miRs needs the Drosha complex, and the ribosomal RNA formation goes through the Nop56 pre-rRNA complex. RNA metabolism involves catabolism, post-transcriptional and epigenetic modifications, as well as repair of RNA. As DEK is reportedly part of the splicing machinery [45] and many of the DEK/NUP214 interactors were related to these processes, our results point to the DEK-portion as decisive for this function of DEK/NUP214. Similarly, the localization of DEK/NUP214 in nuclear speckles (or interchromatin granule clusters) can be attributed to its function as these self-assembled nuclear structures are formed of RNA and proteins and seem to be involved in leukemogenesis as they control transcription, splicing, and RNA transport [46].

That RNA biology plays a role in leukemogenesis of t(6;9)-AML is in accordance with recent findings showing that the lncRNA HOTAIRM1 is highly expressed in t(6;9)-AML patients and closely related to a pattern of miR expression, in particular of miR-196b [47,29]. Both HOTAIRM1 and miR196b represent individual and independent prognostic factors. The expression of the lncRNA HOTAIRM1 and different miRs, particularly miR-196b, depends on DEK/NUP214. As this was also the case in SET/NUP214 positive cells, it has been speculated that binding the NUP214 portion of the fusion protein to the specific HOXA genes would result in an up-regulation of HOTAIRM1 [29]. This would imply that NUP214, a member of the nuclear pore complex, can exhibit features of a transcription factor. NUP214 is frequently involved in other fusions, such as SET/NUP214 or NUP214/ABL1 [42]. SET/NUP214 and DEK/NUP214 share the identical portion of NUP214. However, the NUP214 portion of NUP214/ABL1 is physically and functionally different [48]. SET and DEK are functionally related [49], which can explain the features SET/NUP214 and DEK/NUP21 share.

Interestingly, also NUP214 is involved in RNA biology. Its interaction with CRM1 interferes with RNA transport and has been established as a therapeutic target [35]. We show that CRM1 binds to all biologically dead DEK/NUP214 mutants, excluding the NUP214-CRM1 interaction and the CRM1 sequester relevant for the leukemogenesis of t(6;9)-AML. Whether the up-regulation of HOTAIRM1 and miRs is due to an aberrant RNA regulation most likely mediated by the DEK portion or a promoter activation by the NUP214 portion has to be further clarified. Our data indicate that the aberrant RNA biology in cells expressing DEK/NUP214 can be attributed to both NUP214 and DEK.

The analysis of biologically dead mutants evidenced functional domains in DEK/NUP214 indispensable for its leukemogenic potential and provides opportunities for developing novel targeted therapy approaches. Putative GSK3β phosphorylation is not only relevant to the functionality of DEK [39] but also to the leukemogenic potential of DEK/NUP214. The axis GSK3β-DEK/NUP214 is essential as a therapeutic target, considering the role of GSK3β in regulating WNT and mTOR/AKT signaling [50].

Also, helix 3 of the NUP214 CC-domain is indispensable for the leukemogenic activity of DEK/NUP214. Most likely, it is not an oligomerization interface like the CC-domains in other fusion proteins, such as BCR/ABL1 or PML/RARα, but a protein-protein interaction domain [42].

The data presented here provide new insights into the leukemogenic mechanisms of DEK/NUP214 and show the enormous potential of bioinformatics and systems biology in determining biological processes and their complexity required to mediate a phenotype and to cause disease.

## Supporting information

S1 Fig The leukemogenic potential of DEK/NUP214 and the TAP-tagged DEK/NUP214. **A.** The immunoblot shows the expression of TAP-tagged DEK/NUP214 in comparison to HA-tagged DEK/NUP214 in 293T cells (α-DEK staining). **B.** Leukemogenic potential of HSPCs transduced with the TAP-tagged DEK/NUP214; PML/RARα - control. The detection of GFP assessed transduction efficiency. Leukemogenic potential assessed by CFU-S12. TAP–empty vector.
(PDF)

S2 Fig The entire complex is built up by the interactome of the DEK/NUP214. MIST analysis of DEK/NUP214 interactors applying a threshold of Log2 fold change corresponding to an increase of interaction of 1.5 fold and a p<0.05. The first shell of their interactors is given to show the complexity of the networks in which DEK/NUP214 is involved.
(PDF)

S3 Fig The Leukemogenic potential of DEK/NUP214 mutants. **A.** Putative GSK3-, PKC-, and CKII-phosphorylation sites and modular organization of DEK. **B.** DEK and DEK/NUP214 and their phosphorylation mutants (not in scale). **C.** Retroviral construct and expression control for DEK and DEK/NUP214 in 293T cells. **D.** Phosphorylation levels of DEK/NUP214 and its phosphorylation mutants. **E.** I-Tasser 3D analysis of the DEK/NUP214 coiled-coil domain revealing five helices and modular organization of DEK/NUP214’s helix-mutants (not in scale). **F.** Influence of the mutations on the leukemogenic potential of DEK/NUP214 in murine HSPCs. 1x10^4^ cells Sca1+/lin- BM cells were transduced with the indicated constructs and maintained for seven days in liquid culture. And then inoculated into lethally irradiated recipients that were culled on day 12, and spleen colonies were counted. The number of colonies in the spleens (n = 3). One representative experiment of three performed that yielded similar results is given (+/- SEM).
(PDF)

S4 Fig Colocalization between DEK/NUP214 and its phosphorylation mutants by CLSM. **A.** Phaco–phase contrast; green fluorochrome–GFP-DEK/NUP214; red fluorochrome–Alexa 594-conjugated secondary Ab detecting the anti-HA Ab. **B.** Determination of DEK, DEK/NUP214, and their phosphorylation mutants in the nuclear fraction of 32D cells obtained by different salt concentrations.
(PDF)

S5 Fig Bioinformatic analysis of biological processes of interactors of DEK/NUP214 and its mutants in g:Profiler and Cytoscape. g:Profiler–Shown are the numbers of the statistically relevant (p≤0.05) processes in the different resources of g:Profiler (GO: MF, GO: BP, KEGG, REACTOME, WP, TF, miRNA, HPA, CORUM, and HP). The further refinement of the g:Profiler output by Cytoscape/EnrichmentMAP is given in Fig 5.
(PDF)

S1 Data Supplementary Material and Methods. (PDF)

S1 Table List of the DEK/NUP214 interactors. (XLSX)

S2 Table MIST analysis of the protein complexes formed by DEK/NUP214 interactors. (XLSX)

S3 Table g:Profiler and Cytoscape analysis of DEK/NUP214 interactors. (CSV)

S4 Table g:Profiler and Cytoscape analysis of DEK/NUP214 versus its mutants. (CSV)

S5 Table GSEA and Cytoscape analysis of DEK/NUP214 versus its mutants. (CSV)

S6 Table Imaris parameter of colocalization between CLU and DEK/NUP214. (XLSX)
