# Supplementary figures and images for: Understanding a high-risk acute myeloid leukemia by analyzing the interactome of its major driver mutation
Source: PLoS Genet. 2022 Oct 26;18(10):e1010463. doi: 10.1371/journal.pgen.1010463 (PMC9639852; doi:10.1371/journal.pgen.1010463)

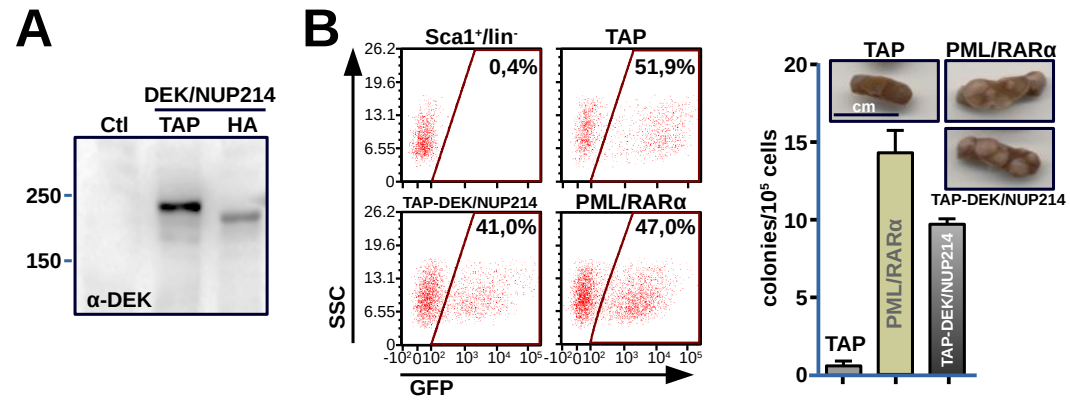

Chiriches et al., S1 Fig

Supplement: S1 Fig — A. The immunoblot shows the expression of TAP-tagged DEK/NUP214 in comparison to HA-tagged DEK/NUP214 in 293T cells (α-DEK staining). B. Leukemogenic potential of HSPCs transduced with the TAP-tagged DEK/NUP214; PML/RARα - control. The detection of GFP assessed transduction efficiency. Leukemogenic potential assessed by CFU-S12. TAP–empty vector. (PDF) [file pgen.1010463.s001.pdf]

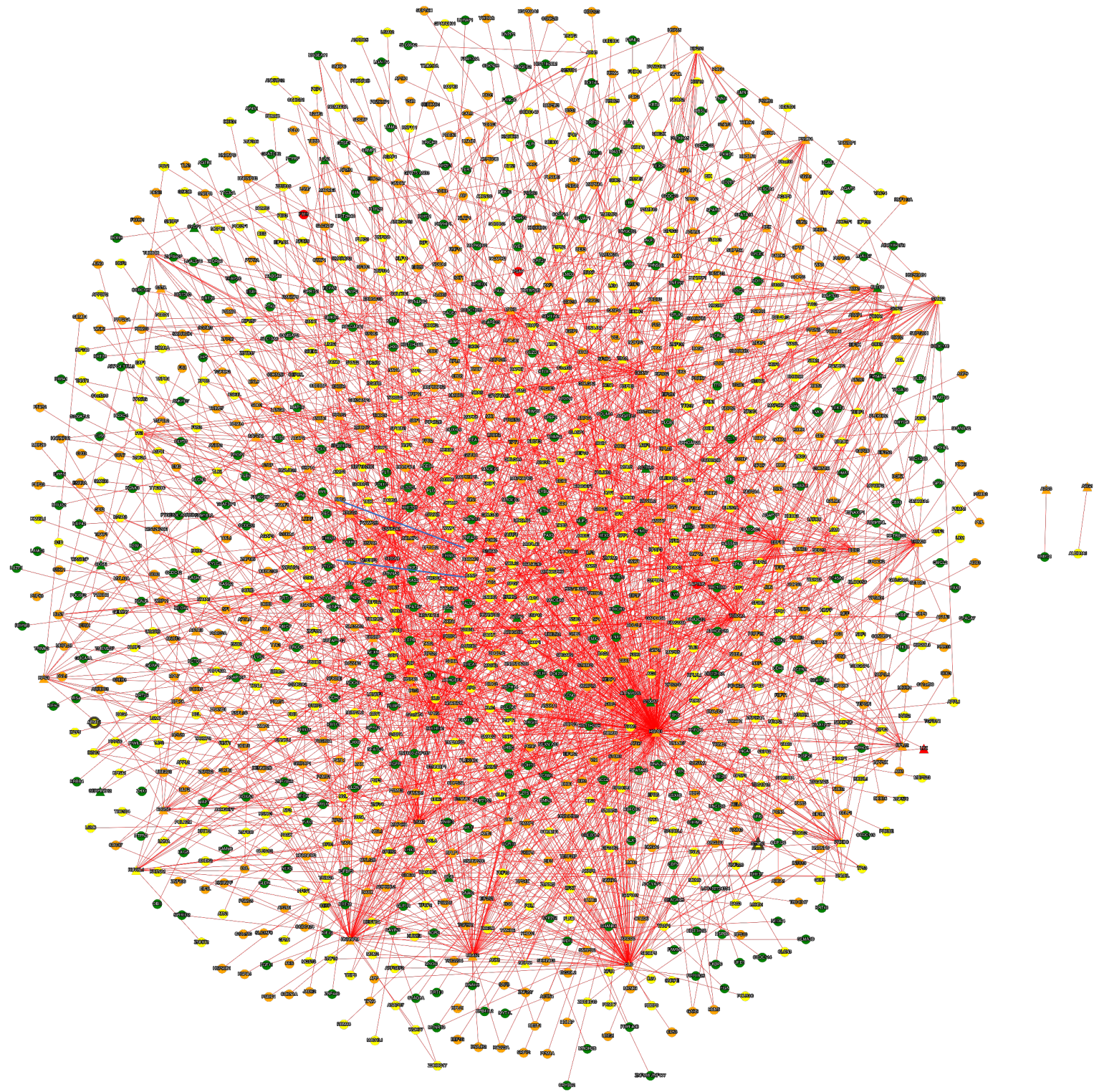

Chiriches et al., S2 Fig

Supplement: S2 Fig — MIST analysis of DEK/NUP214 interactors applying a threshold of Log2 fold change corresponding to an increase of interaction of 1.5 fold and a p<0.05. The first shell of their interactors is given to show the complexity of the networks in which DEK/NUP214 is involved. (PDF) [file pgen.1010463.s002.pdf]

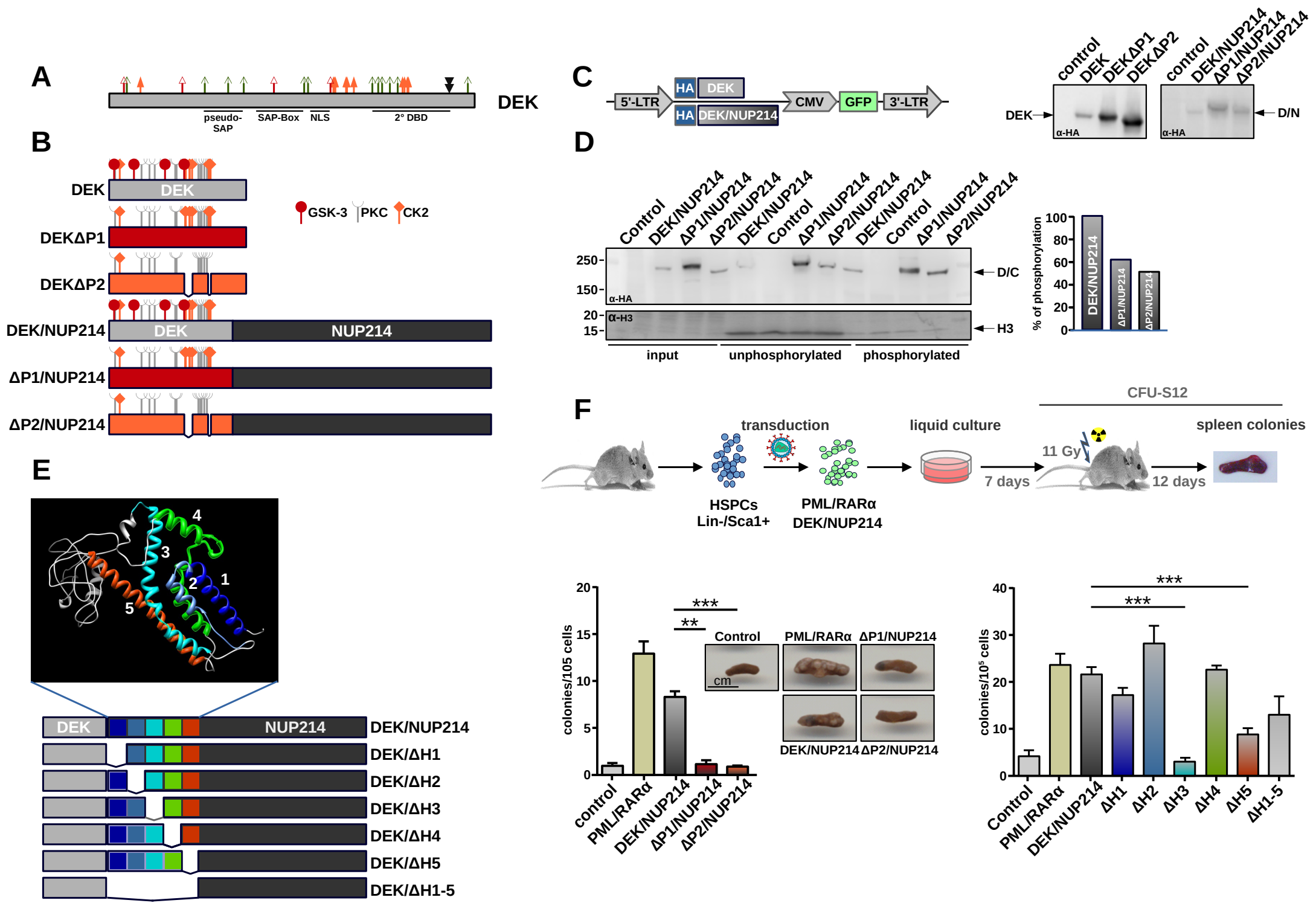

Chiriches et al., S3 Fig

Supplement: S3 Fig — A. Putative GSK3-, PKC-, and CKII-phosphorylation sites and modular organization of DEK. B. DEK and DEK/NUP214 and their phosphorylation mutants (not in scale). C. Retroviral construct and expression control for DEK and DEK/NUP214 in 293T cells. D. Phosphorylation levels of DEK/NUP214 and its phosphorylation mutants. E. I-Tasser 3D analysis of the DEK/NUP214 coiled-coil domain revealing five helices and modular organization of DEK/NUP214’s helix-mutants (not in scale). F. Influence of the mutations on the leukemogenic potential of DEK/NUP214 in murine HSPCs. 1x10^4^ cells Sca1+/lin- BM cells were transduced with the indicated constructs and maintained for seven days in liquid culture. And then inoculated into lethally irradiated recipients that were culled on day 12, and spleen colonies were counted. The number of colonies in the spleens (n = 3). One representative experiment of three performed that yielded similar results is given (+/- SEM). (PDF) [file pgen.1010463.s003.pdf]

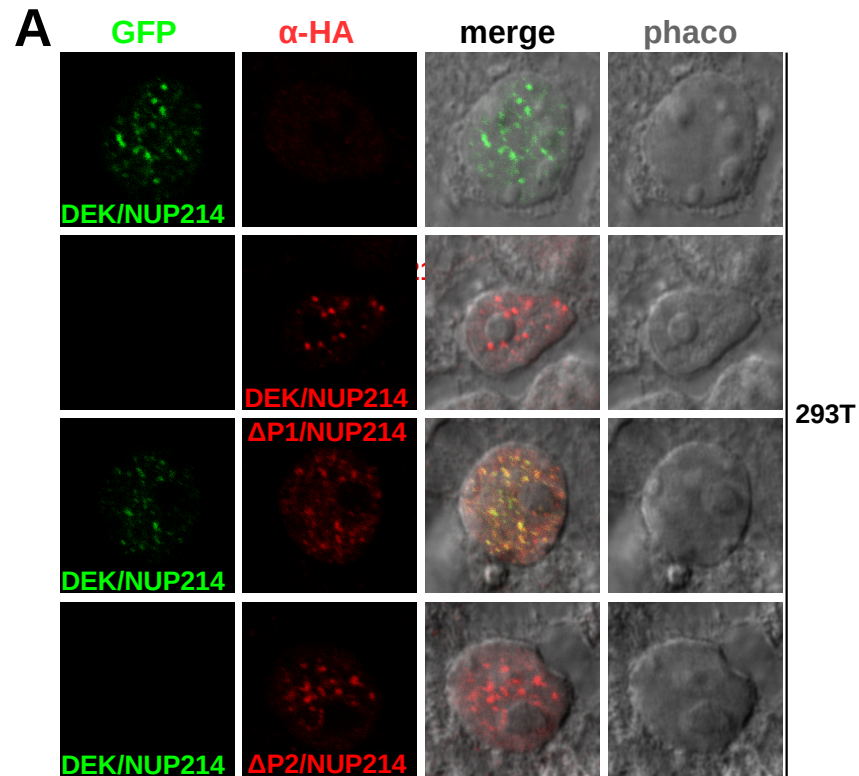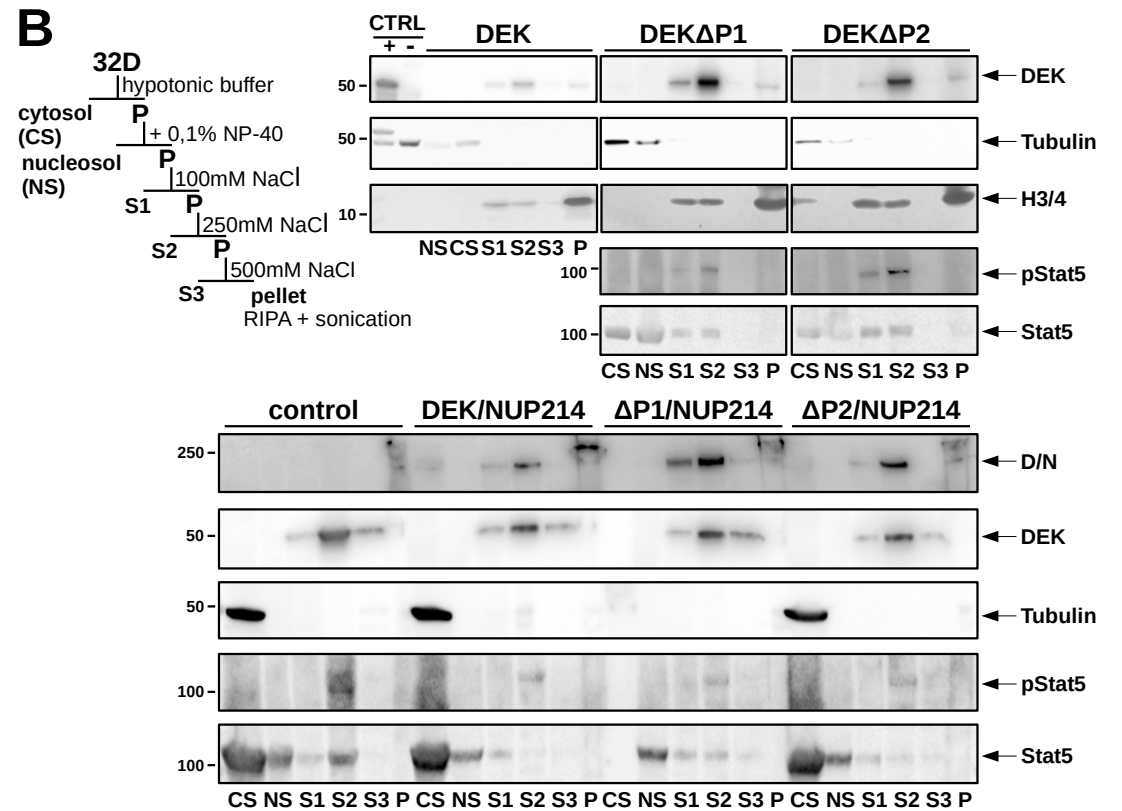

Supplement: S4 Fig — A. Phaco–phase contrast; green fluorochrome–GFP-DEK/NUP214; red fluorochrome–Alexa 594-conjugated secondary Ab detecting the anti-HA Ab. B. Determination of DEK, DEK/NUP214, and their phosphorylation mutants in the nuclear fraction of 32D cells obtained by different salt concentrations. (PDF) [file pgen.1010463.s004.pdf]

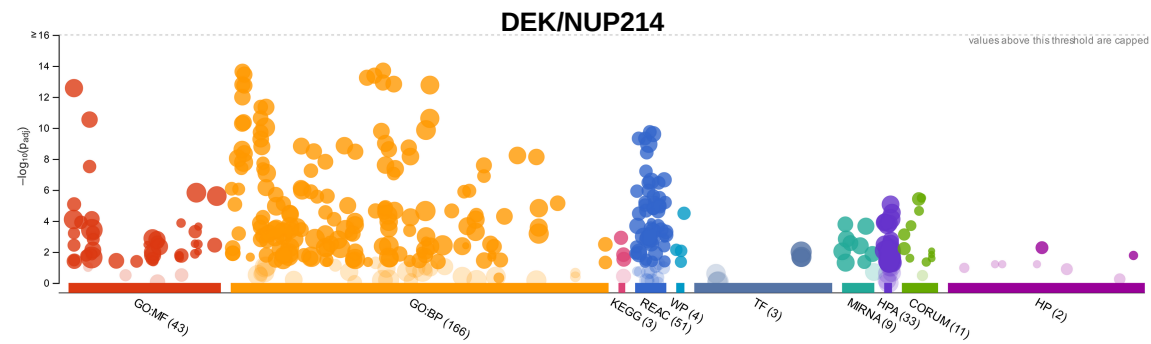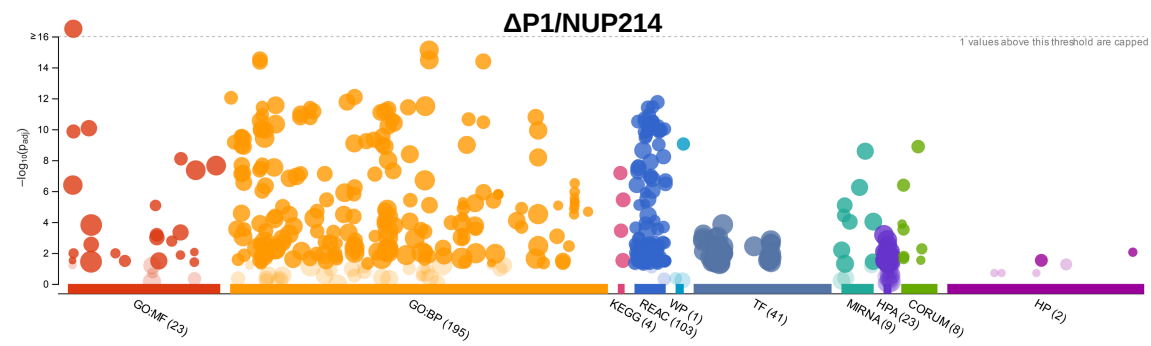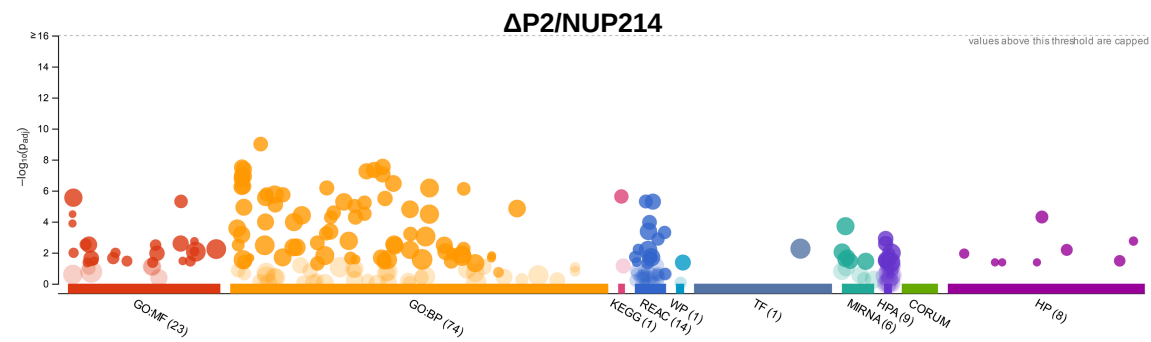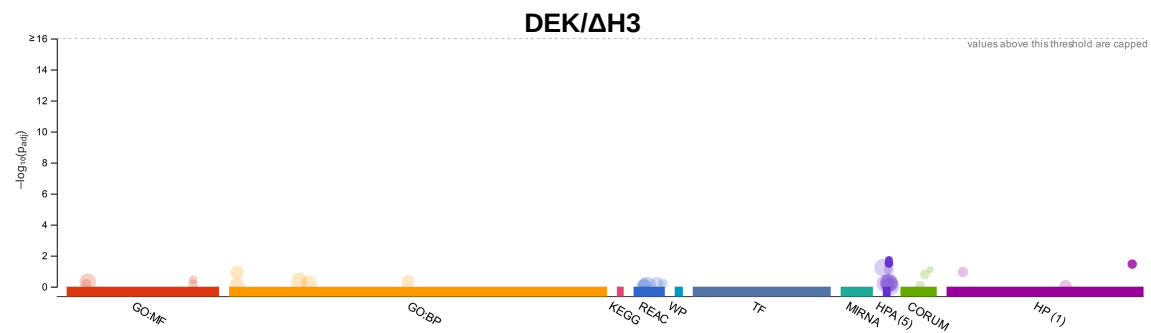

Supplement: S5 Fig — g:Profiler–Shown are the numbers of the statistically relevant (p≤0.05) processes in the different resources of g:Profiler (GO: MF, GO: BP, KEGG, REACTOME, WP, TF, miRNA, HPA, CORUM, and HP). The further refinement of the g:Profiler output by Cytoscape/EnrichmentMAP is given in Fig 5. (PDF) [file pgen.1010463.s005.pdf]
