# Supplementary material for: Understanding a high-risk acute myeloid leukemia by analyzing the interactome of its major driver mutation
Source: PLoS Genet. 2022 Oct 26;18(10):e1010463. doi: 10.1371/journal.pgen.1010463 (PMC9639852; doi:10.1371/journal.pgen.1010463)
Supplement: S1 Data — (PDF) [file pgen.1010463.s006.pdf]

Understanding a High Risk Acute Myeloid Leukemia by Analyzing the Interactome of Its Major Driver Mutation

CLAUDIA CHIRICHES[1,2,*,#], NATHALIE NICOLAISEN[3,#], MARIA WIESKE[3,#], HEBA ELHADDAD[1,2,4], ECMEL MEHMETBEYOGLU[1,2], CAROLINE ALVARES[1,2], DÖRTE BECHER[5], PAUL HOLE[1,2], OLIVER GERHARD OTTMANN[1,2], and MARTIN RUTHARDT[1,2,*]

Supplementary Materials and Methods

Further information and requests for resources and reagents should be directed to and will be fulfilled by the Lead Contact, Claudia Chiriches.

I-Tasser. I-TASSER (Iterative Threading ASSEmbly Refinement)(https://zhanglab.comb.med.umich.edu/I-TASSER) was used to generate the hypothetic 3D structure of the coiled-coil domain in NUP214 based on its aa. sequence (Zhang, 2008). In a first step („threading") the aa. sequence was compared to sequences present in the Protein Data Bank (PDB)(https://www.ebi.ac.uk/pdbe/)(Burley et al., 2017) for which the 3D structure is known. In a second step („assembly") the resulting structure fragments were assembled to create hypothetic 3D models. The energetically most favorable models were then analyzed in a last step („refinement") for the presence of possible hydrogen-bonds and the most favorable backbone torsions simulations were calculated.

Plasmids, Oligos. The DEK/NUP214 helix mutants were obtained using the Cold Fusion Cloning Kit (System Biosciences) using the following primers (5'-3') DC Helix1-fw GCC AGT GCT AAC TTG GAT GTG AAT GAT GTT; DC Helix1-rev AAC ATC ATT CAC ATC CAA GTT AGC ACT GGC; DC Helix2-fw AAA TTT GCT GTC CAA CAA AGG CACG TGC TT; DC Helix2-rev AAG CAG GTG CCT TTG TTG GAC AGC AAA TTT; DC Helix3-fw CTG CTT GTG CCA GAG AGC CTG TCC TCG GCT; DC Helix3-rev AGC CGA GGA CAG GCT CTC TGG CAC AAG CAG; DC Helix4-fw AGT TTT GAC AGT GAC AAG ACC CCA CCA GTG; DC Helix4-rev CAC TGG TGG GGT CTT GTC ACT GTC AAA ACT; DC Helix5-fw GTG AGA TCC ACT GCT ACG TCC TGT AAA GAT; DC Helix5-rev ATC TTT ACA GGA CGT AGC AGT GGA TCT CAC. Point mutations in the putative GSK3β phosphorylation sites were introduced by site directed mutagenesis using the QuikChange Site Directed Mutagenesis Kit (Agilent) according to the manufacturer's instructions. The following primers

were used: (5'-3') DEK S30A-fw GTC CCA GAG AGG AGG CCG AGG AGG AAG AGG; DEK S30A-rev CCT CTT CCT CCT CGG CCT CCT CTC TGG GAC AAG CTG; DEK 240/241-rev CAG CTT CTT TAT CTT CAT CAT CTG CAG CCT CTT CCT TGT TTT TCT TTT C; DEK-A40G-fw AGG GAA CCC CCG CCC AGC CCG CG; DEK-A40G-rev CGC GGG CTG GGC GGG GTT CCC T; DEK-A196G-fw AAG AAA AAG TAG AGA GGT TGG CAA TGC AAG TCT CTT CCT TAC; DEK-A196G-rev GTA AGG AAG AGA CTT GCA TTG CCA ACC TCT CTA CTT TTT TCT T; DEK-T502G-fw GAG GTT CTT GAT TTG GAG AGA GCA GGT GTA AAT AGT GAA CTAG; DEK-T502G-rev CTA GTT CAC TAT TTA CAC CTG CTCT CTC CAA ATC AAG AAC CTC; DEK-T676G-fw ACC AAA TGT CCT GAA ATT CTG GCA GAT GAA TCT AGT AGT GAT G; DEK-T676G-rev CAT CAC TAC TAG ATT CAT CTG CCA GAA TTT CAG GAC ATT TGG T. The sequences were then transferred into the pEntry vector (Gateway – Thermo Scientific, Darmstadt Germany) for further recombinations into destinations vectors. The TAP-TAG DEK/NUP214 constructs were generated by transferring a TAP-tag sequence from a TAP-tag-pUC19 (kindly provided by Elena Puccetti, Institute for Molecular Biology and Tumor Research, Philipps University, Marburg, Germany) in frame with the sequences encoding the mutants in pEntry by using the Cold Fusion cloning kit (System Biosciences,). The correctness of sequence was confirmed for all constructs by Sanger sequencing.

Antibodies. The following antibodies were used: anti-DEK mouse monoclonal (BD Biosciences Cat# 610948), anti-NUP214 rabbit polyclonal (Abcam Cat# ab70497), anti-HA.11 (16B12) mouse monoclonal (Covance Cat# MMS-101P-200), anti-H3K9me3 rabbit polyclonal (Millipore Cat# 07-442), anti-STAT5 (C-17) rabbit polyclonal (Santa Cruz Biotechnology Cat# sc-835), anti-phospho STAT5 A/B (Y694/699) (clone 8-5-2) mouse monoclonal (Millipore Cat# 05-495), anti-Tubulin alpha (DMA1) mouse monoclonal (Lab Vision Cat# MS-581-P), anti-CLU rabbit polyclonal (Santa Cruz Biotechnology Cat#H-330), anti-H3 (clone 3H1) rabbit monoclonal (Cell Signaling Technology Cat#9717), and anti H4 rabbit polyclonal (Millipore Cat# 07-108).

Nuclear and cytoplasmic fractionation. After the procedure described for the chromatin precipitation the different compartments were extracted by incubation in increasing NaCl concentrations followed by centrifugation and the suspension of the resulting pellet in RIPA-buffer by as described by Kappes et al, 2001. After methanol-chloroform precipitation the cells were loaded on a SDS-PAGE for immunoblotting.

Enrichment of phosphorylated proteins. For the enrichment of threonin- and lysin-phosphorylated proteins from 1x10^7cells the Qiagen PhosphoProtein Purification" Kit was used according to the manufacturer's instructions (Qiagen). Unphosphorylated proteins were precipitated by methanol-chloroform-precipitation from the eluate. 50µg of phosphorylated and unphosphorylated proteins of each sample, respectively, were loaded on SDS-PAGE for immunodetection.

In-Gel Detection of phosphorylated proteins. For In-Gel Detection of phosphorylated proteins, the Pro-Q Diamond stain (Thermo-Fisher) was used according to the manufacturer's instructions. Briefly, the SDS-PAGE

gels were soaked twice for 30 min. in ProQ Fixation (50% methanol plus 10% acetic acid), after intense washing in H_2O, the gels then were stained at RT for 70 min. protected from light. For destaining after intense washing in H_2O the gels were soaked in 20% acetonitril in 50mM sodium acetate (pH 4.0). The bands were detected at 600mm using the Odyssey Fc Imaging Systems (Li-Cor Biotechnologies).
